# Magnetic field control with dual robotic tunable magnetic end effectors

**DOI:** 10.1038/s44172-026-00629-0

**Published:** 2026-03-04

**Authors:** Kiana Abolfathi, Jiacheng Zhu, James H. Chandler, Keyoumars Ashkan, Pietro Valdastri, Hongsoo Choi, Xiaojun Zhai, Ali Kafash Hoshiar

**Affiliations:** 1 School of Computer Science and Electronic Engineering, University of Essex, Colchester, UK; 2 STORM Lab, Institute of Robotics, Autonomous Systems and Sensing, School of Electronic and Electrical Engineering, University of Leeds, Leeds, UK; 3 Department of Neurosurgery, King’s College Hospital, London, UK; 4 DGIST-ETH Microrobotics Research Center, Daegu Gyeongbuk Institute of Science and Technology (DGIST), Daegu, Republic of Korea; 5 Department of Biomedical Engineering, Institute for Applied Life Sciences, University of Massachusetts Amherst, Amherst, MA USA

**Keywords:** Mechanical engineering, Biomedical engineering

## Abstract

Magnetic manipulation is increasingly used in medical applications for its potential in remote control. However, precise magnetic field generation in large workspaces remains challenging. This paper introduces an adaptive robotic end effector, the tunable magnetic end effector (TME), capable of generating spatially controllable magnetic fields. By integrating permanent magnets, the TME enables accurate magnetic control for wireless manipulation of miniaturized medical devices. Compared to standard switchable permanent magnets, TME offers enhanced field control suited for delicate operations. Finite element (FEM) simulations and experiments confirm reliable ON/OFF field switching, showing a 7.2% average error. Key design parameters (magnet size, material, and arrangement) were optimized via simulation. An artificial neural network (ANN), trained on spatial, rotational, and magnetic data, enables adaptive control. Proof-of-concept demos include steering millimeter-scale magnetic carriers, shaping magnetic soft robots, and directing magnetic nanoparticle swarms. The dual-TME configuration further expands the effective manipulation workspace and enables dynamic switching of magnetic field directions across different regions, thereby enhancing the system’s applicability.

## Abstract (web summary)

Magnetic microrobot control typically trades off between the simplicity of permanent magnets and the controllability of electromagnetic systems. Kiana Abolfathi and colleagues introduce a tunable magnetic end effector that combines permanent magnets with switchable, spatially controllable magnetic fields for precise medical manipulation.

## Introduction

Magnetic manipulation is widely used to wirelessly actuate and control small-scale medical devices^1,2^, including soft tethered robots^3–5^, untethered magnetic robots^6–8^, and microswarms^9,10^. This technology enables precise robotic navigation and manipulation within the body, facilitating advanced medical procedures and treatments, such as minimally invasive surgery^11^, targeted drug delivery^12,13^, and precision diagnostics^14^. Current approaches to magnetic actuation primarily utilize two techniques: electromagnetic actuation (EMA) and robotically controlled permanent magnet (PM) systems, each offering distinct advantages for specific applications^15^.

EMA employs electromagnets to generate controlled magnetic fields. Controlling the coil current allows for real-time magnetic field adjustment and therefore precise manipulation^16,17^, especially within complex environments^18^. EMA has shown significant promise in applications such as targeted drug or stem cell delivery^19,20^ and intravascular interventions^21^. Studies demonstrate that the EMA has potential for precise manipulation and remote control of microrobots^21,22^. Although EMA demonstrates high capabilities for clinical applications^15,21^, its implementation faces challenges, due to the required complex and bulky electromagnetic setups^23,24^.

**Fig. 1:**
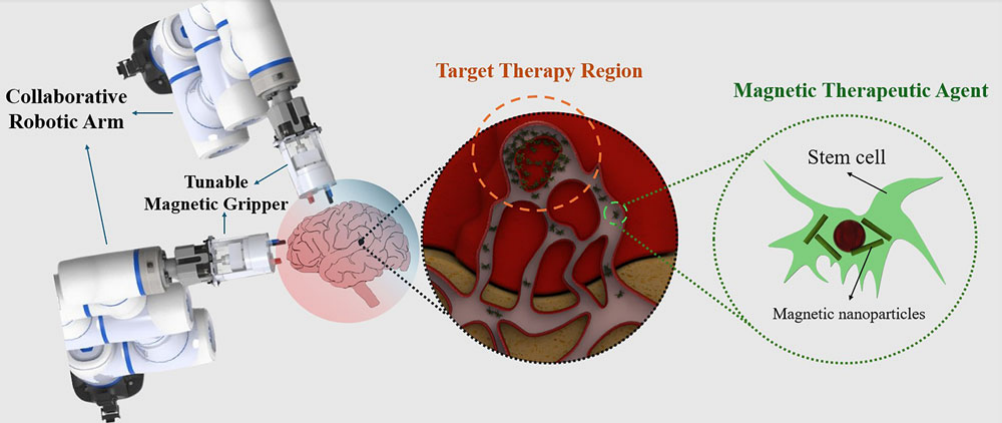
Robotics setup for controlling PMs in targeted medical applications.

As an alternative to EMAs, robotically controlled PM systems can provide a more compact, and flexible platform to deliver a more expansive active workspace for magnetic manipulation at clinically relevant scales. A collaborative magnetic manipulation approach is introduced, employing two robotically actuated PMs to achieve precise and programmable control of magnetic objects in three-dimensional space. Furthermore, a trajectory planning method has been developed to coordinate the motion of the magnets, enabling accurate magnetic actuation tailored to medical applications^25,26^. Integrating PMs into robotic systems enables precise manipulation of magnetic fields through the robotic arm’s mechanical movements, facilitating wireless control of objects (shown in Fig. 1).

PM-based methods offer several inherent benefits, including (1) stronger magnetic fields at similar scales, (2) eliminating overheating risks^27^, (3) structural simplicity, and (4) enhanced workspace flexibility. PMs allow for easier adjustment of the workspace by physically repositioning the magnets, which is particularly valuable for applications requiring flexible spatial configurations^28,29^. Although these systems are advantageous for clinical applications due to their ease of use, their functionality can be constrained by their reliance on robotic arm movement.

While several studies have proposed switchable electropermanent magnets^30^ and PM for specific applications, such as climbing robots^31,32^, and various industrial and robotic contexts^33–35^, none have effectively considered their utility for precise magnetic field control in different regions of the workspace. The development of a tunable magnetic end effector (TME) capable of manipulating magnetic medical devices (such as magnetic carriers and intravascular devices) remains critical.

In this study, we advance PM-based magnetic manipulation by introducing a tunable end effector capable of dynamically adjusting the magnetic field and gradient. This design enables precise magnetic control across a flexible and movable workspace (illustrated conceptually in Fig. 1), which is supported by a data-driven field generation scheme. The key contributions of this work are as follows:

1. Introducing a tunable PM-based end effector capable of generating variable magnetic fields, including ON/OFF states, specifically for medical applications.
2. Enabling magnetic field direction control in different regions through two collaborative robots equipped with TMEs.
3. Developing a data-driven approach for generating a magnetic field.

The paper is organized as follows: First, an artificial neural network (ANN) is trained on a comprehensive dataset, achieving a high prediction accuracy for magnetic field control. Next, a proof-of-concept study validates the system’s key contributions, demonstrating its effectiveness in achieving tunable multi-region magnetic control. Finally, we introduce the design concept underlying the development of TME. Subsequently, we employ modeling and simulation to assess the design parameters and validate the results through experimental testing.

## Results

### Experimental investigation of the tunable field

**Fig. 2:**
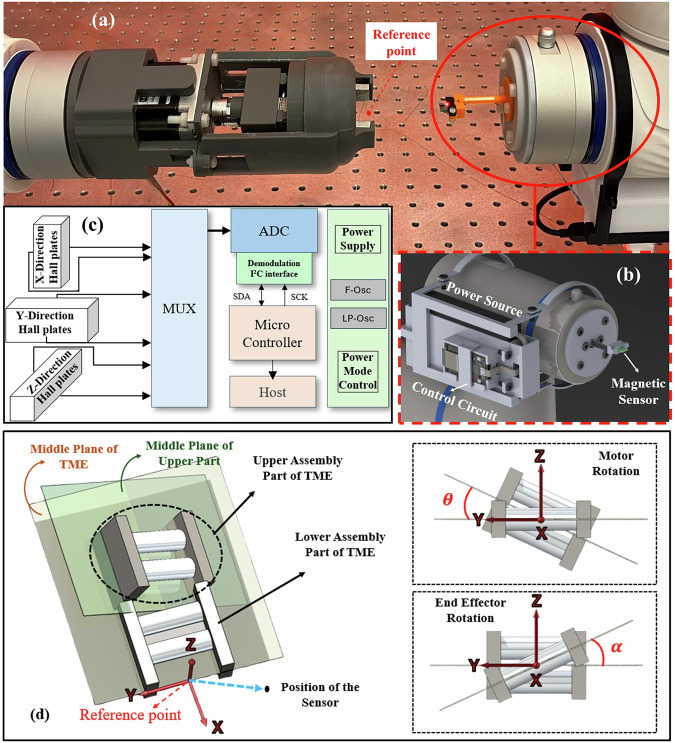
Data collection using the magnetic sensor. **a** Experimental setup for data collection, **b** Designed sensor holder, and **c** The sensor schematic. **d** Schematic showing the distance vector (*r*_*p*_) between the TME and the magnetic sensor, along with the motor rotation angle (θ) and the end effector angle (α).

To investigate the magnetic field generated by the designed TME, a movable sensor system was developed for data collection. As shown in Fig. 2, the system comprises a power source, control circuit, and magnetic sensor, all securely mounted on a holder for attachment to the robotic arm. A compact system for measuring three-axis magnetic field intensity was developed using the TLE493D 3D Hall Effect sensor and an ESP32 microcontroller. The TLE493D, with a resolution of 65 µT, enables precise detection of small magnetic field variations. A low-ripple power supply module ensures system stability and high measurement accuracy. The data collection process is demonstrated in Supplementary Video S1.

The system integrates a stable power supply, a high-resolution ADC, and a control unit. An embedded program on the ESP32 acquires data from the TLE493D, while the built-in Wi-Fi module enables remote operation (Fig. 2c). Communication between the microcontroller and a host system utilizes a local Wi-Fi network.

The upper part of the TME is capable of 360° continuous rotation at a frequency of 2 Hz, enabling dynamic modulation of the magnetic field. Consequently, ON and OFF switching of the magnetic field can be achieved at an effective frequency of 4 Hz, providing rapid and controllable field actuation for experimental testing.

Additionally, the end effector (Joint 6) of the Dobot CR3 robot used to manipulate the TME can achieve a maximum rotational frequency of 0.5 Hz, as specified by the manufacturer. This motion is not continuous but can be utilized for stepping (incremental or discrete) rotational actuation. Although this frequency is insufficient for generating a rotating magnetic field, it is adequate for the controlled alignment and positioning tasks performed in this study, where magnetic gradient field steering was primarily employed.

### Parameters study

Data collection was based on three key parameters: (1) the location vector from the TME’s zero point to the magnetic sensor (*X*, *Y*, *Z*), (2) the rotational angle of the TME (α), and (3) the stepper motor rotation angle (θ).

**Fig. 3:**
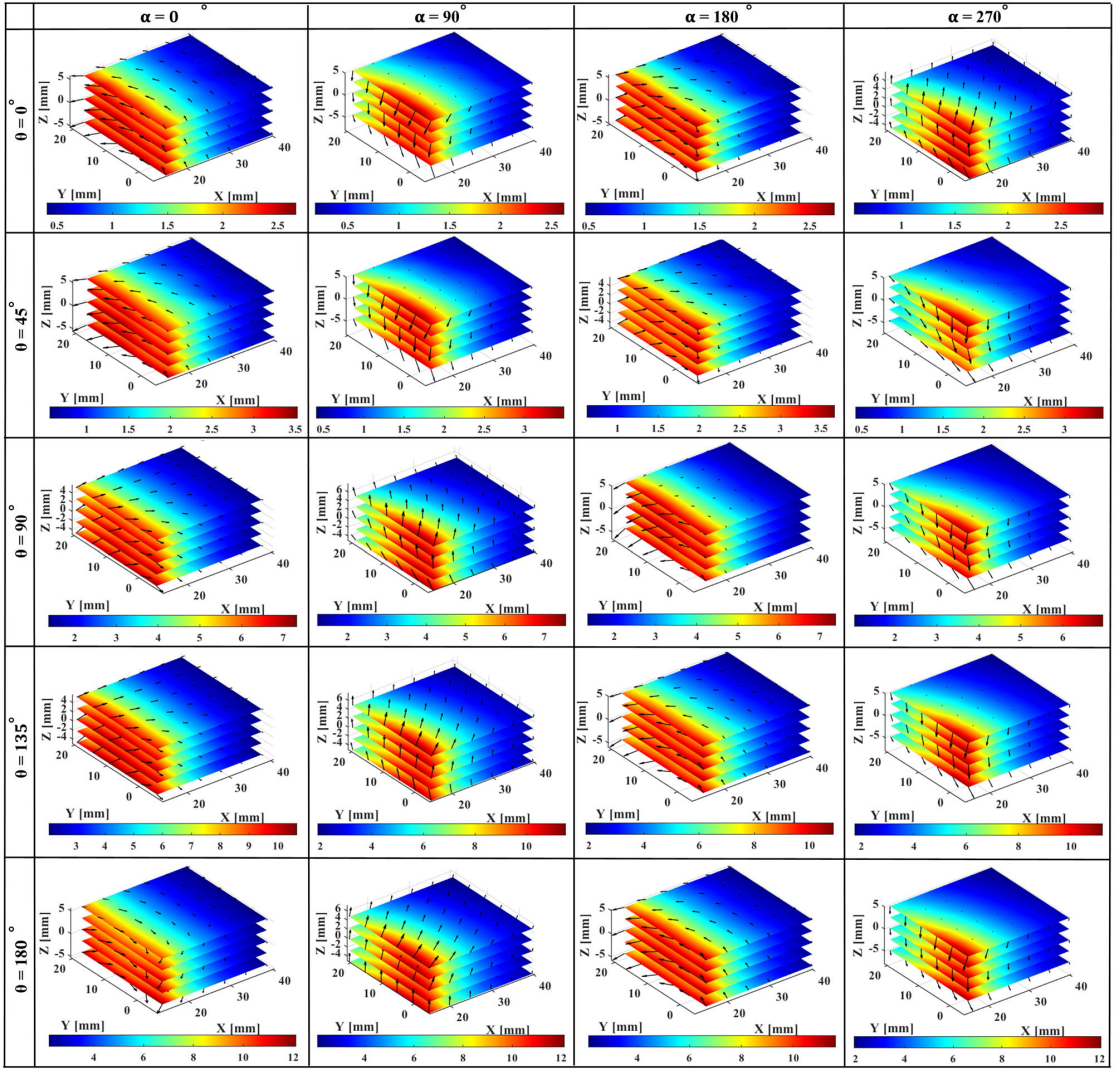
Magnetic field magnitude (mT) in 3D space under different rotation angle conditions for the TME and stepper motor.

The 3D visualization of the magnetic field magnitude is presented in Fig. 3. The results indicate that the magnitude of the magnetic field decreases with increasing distance from the TME in all three directions. However, the rate of this decline, as well as the intervals of variation, differ depending on the stepper motor rotation angle (θ) that will be discussed in the following section.

The results in Fig. 3 show that changing the rotation angle α causes only a slight change in the overall magnitude of the magnetic field under otherwise identical conditions. The key finding of this study, however, is that while the field magnitude remains nearly constant, the direction of the magnetic field changes significantly. This directional variability is particularly valuable for applications requiring precise control, such as small-scale robotics, where sensitivity to magnetic field direction is crucial for accurate manipulation. The collected 3D spatial data illustrating magnetic field variations under OFF and ON conditions is visualized using MATLAB and presented in Supplementary Video S2.

**Fig. 4:**
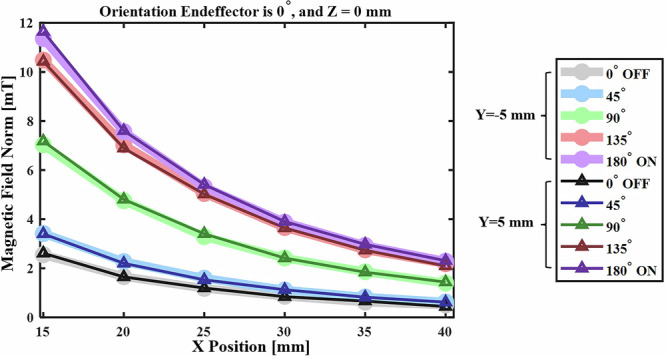
Effect of stepper motor angle (θ) on magnetic field magnitude, showing a tunable magnetic field.

As shown in Fig. 4, variations in the angle θ influence the magnitude of the generated magnetic field. Figure 4 further illustrates the effect of these changes in θ along the *X*-direction, comparing the tunable magnetic field behavior from the OFF position to the ON position. Therefore, this parameter could be used to control the magnitude of the magnetic field generated at a specific point in space without changing the position of the TME. This capability allows precise magnetic field control without physically moving the robotic arm, addressing a key challenge in magnetic field manipulation with robotic systems.

The results demonstrated that, for the given design, the total magnetic field could vary from 0 to 12 mT in the workspace by adjusting the position of the TME within five parameters: (*x*, *y*, *z*, α, θ). By modifying only the position of the TME (*X*, *Y*, *Z*) under the OFF condition, the magnetic field could range from 0 to 2 mT. Additionally, by modifying the parameters α and θ, the direction of the magnetic field can be further adjusted.

**Fig. 5:**
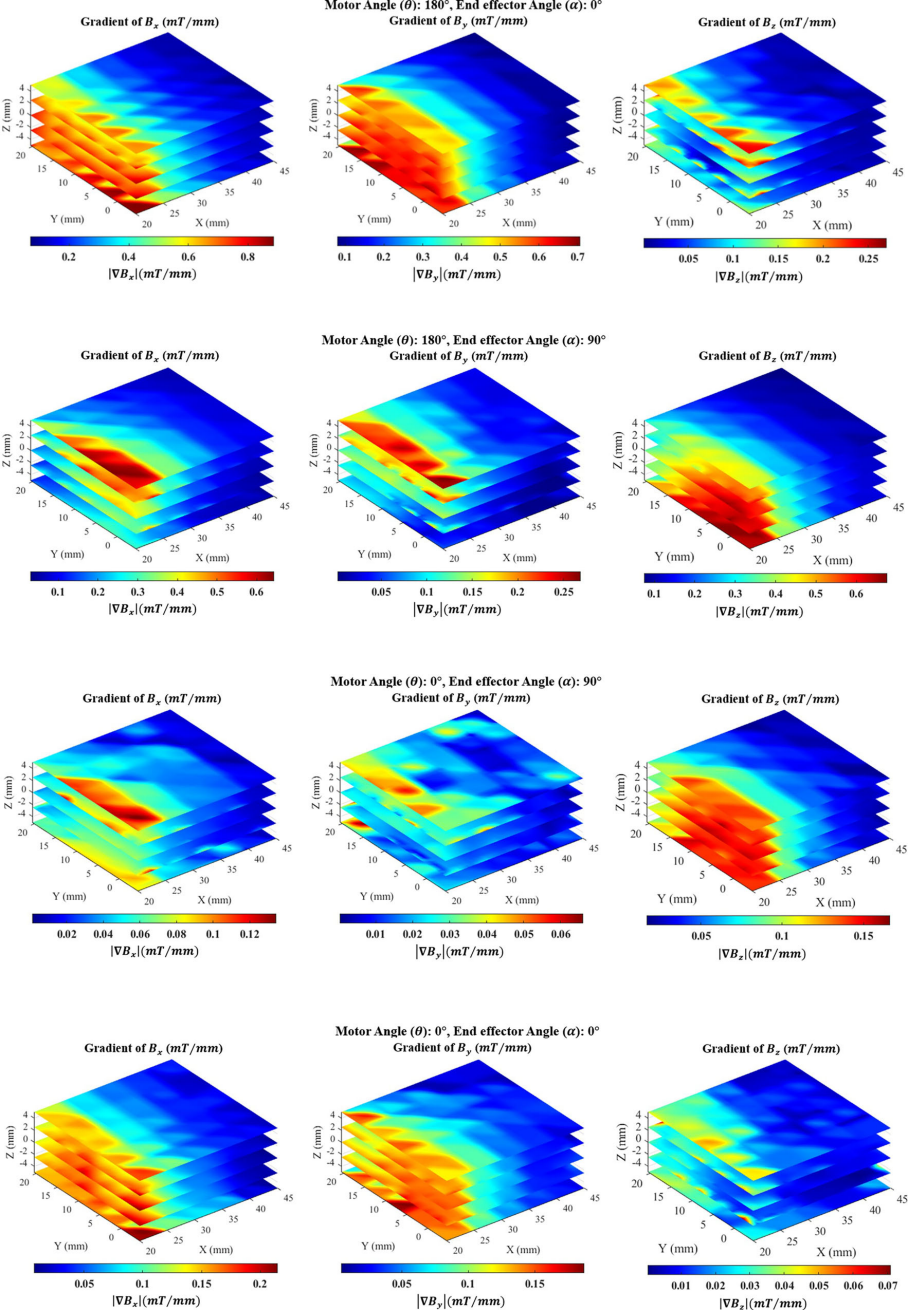
The magnetic field gradient varies along the *X*, *Y*, and *Z* directions under both ON and OFF conditions. This experiment was conducted over three trials, with the TME oriented at 0° and 90°.

Controlling the gradient field is crucial in medical robotics for precise device manipulation in procedures like endovascular interventions. The TME’s ability to dynamically adjust the gradient magnetic field has the potential to enhance accuracy in these applications. The results indicate that decreasing the motor rotation from 180° to 0° leads to a decrease in the gradient field. The variations in the gradient field for the two operational states of the TME, OFF and ON, are presented in Fig. 5.

Based on these investigations (Fig. 5), the fabricated TME is capable of generating magnetic fields ranging from 0 to 12 mT within the workspace, with a corresponding magnetic field gradient of 0 to 0.86 T/m. The complete magnetic actuation system combines a collaborative robotic arm with the TME, providing a total of seven degrees of freedom (DOF): six from the robotic arm itself and one additional redundant DOF from the internal motor rotation within the TME structure. This additional DOF enables direct control of the magnetic field generation, beyond the arm’s physical movement.

In PM-based robotic manipulators, the workspace is governed by arm reach, unlike coil-based systems with geometry-defined limits. In this work, a CR3 collaborative robot was employed, offering a 620 mm reach (spherical-cap volume considering joint limits). Within this region, the TME can be freely oriented in six DOF, allowing precise control of the magnetic field direction. This results in an effective cubic workspace of approximately 45 × 45 × 45 mm in which the magnetic field can be dynamically tuned.

When compared with existing systems, the proposed TME demonstrates unique advantages. Helmholtz and Maxwell coils, for instance, typically provide homogeneous fields within workspaces of about 10–20 cm³, but they are bulky, fixed in geometry, and lack reconfigurability. Their gradients are relatively low, generally below 0.3–0.5 T/m, whereas the TME achieves up to 0.86 T/m within a reconfigurable workspace defined by the robotic arm’s reach. Unlike coils, which require rigid structures, the TME benefits from portability and the ability to adjust its position and orientation dynamically.

Relative to other permanent-magnet-based robotic manipulators, the proposed system offers complementary strengths. Prior works have demonstrated higher absolute magnetic field magnitudes, often exceeding 30–200 mT^25,28,36^, and in some cases, much larger workspaces. However, these systems are often limited in DOF (typically 3–6 DOF) and lack the ability to selectively deactivate or modulate the magnetic field. For instance, rotating PM systems can generate high gradients (up to 7 T/m)^37^ but are constrained to fixed geometries and limited spatial coverage. Similarly, compact permanent-magnet manipulators provide high localized forces but with restricted workspace dimensions, often within a few centimeters.

In contrast, the dual TME integrated with the CR3 robot provides a balance between workspace size, gradient strength, and controllability. While its maximum field strength (12 mT) is lower than that of some coil or PM systems, its reconfigurability, seven DOFs, and ON/OFF control enable more flexible and precise manipulation in localized regions. This balance highlights the value of the proposed design for medical robotics applications, such as endoscopy and minimally invasive interventions, where dynamic control and localized high gradients are more critical than a large uniform field.

A comparative summary of the proposed TME system with existing permanent-magnet-based platforms is provided in Table S1 of the Supplementary Materials. While some reported systems achieve higher magnetic field magnitudes (30–94 mT) or forces, they typically suffer from limited controllability, fewer DOF, and fixed geometries. In contrast, the dual-TME configuration integrated with the CR3 robotic arm offers a balanced performance, achieving fields up to 12 mT, gradients of 0.86 T/m, and seven DOF with rapid response times (<150 ms). These results underscore the TME’s versatility, compactness, and suitability for precise microrobotic manipulation across various applications. Moreover, Table S2 of the Supplementary Materials demonstrates a comparison between the functionality of the proposed dual TME actuation system and several prior PM-based actuation platforms. The results show that the dual TME provides distinct functional advantages, such as switchable ON/OFF states, independent field modulation, and multi-region magnetic field control that are not achievable with previous systems.

### Dataset and neural network model

A comprehensive dataset using the experimental setup presented in Fig. 2 was developed, consisting of 120,000 unique conditions that span a wide range of configurations. The dataset captures variations in the TME’s spatial positions along three axes (*x*, *y*, *z*), its rotational states (ranging from 0° to 359°), and the stepper motor’s discrete rotational settings (0°, 45°, 90°, 135°, 180°). Additionally, the magnetic field characteristics, including the field’s magnitude in three orientations relative to the *x*, *y*, and *z*-axes, were recorded. This comprehensive data set serves as a foundation for modeling the nonlinear relationships between these input parameters and their corresponding system outputs.

To analyze and predict the system’s behavior, an ANN was developed. The network architecture consists of an input layer, three hidden layers with five neurons each, and an output layer representing the target parameters. Each hidden layer employs a ReLU activation function to capture nonlinearities, and the training is carried out using the Levenberg–Marquardt algorithm with a maximum of 1000 epochs and a mean squared error goal of 1 × 10⁻⁵. The dataset was divided into 80% for training and 20% for testing using stratified cross-validation to ensure balanced representation of categorical motor rotation states. The inputs to the ANN include magnetic field magnitude and angular orientation relative to the *x*-axis, while the outputs are the motor’s categorical rotation state, the TME’s normalized rotation, and the normalized *x*, *y*, and *z* positions of the end effector. To generate final predictions, the categorical motor states are decoded back into their physical rotation angles, the TME rotation is rescaled to degrees, and the predicted positions are denormalized to millimeters. This workflow ensures that the outputs of the ANN are interpretable in physical terms and directly comparable to experimental measurements. The steps of this algorithm are summarized in Table 1.

**Table 1:** ANN algorithm for data magnetic field control

	Step	Description
1	Dataset	Load data containing stepper motor rotations, TME rotation, robotic arm position (*X*, *Y*, *Z*)
2	Data processing	- Normalized position to [0,1]: $X:\frac{\left(x,|,{x}_{\min }\right)}{{x}_{\max }},Y:\frac{\left(y,|,{y}_{\min }\right)}{{y}_{\max }},Z:\frac{\left(z,|,{z}_{\min }\right)}{{z}_{\max }}$ - TME rotation scaled to [0,1]: mod(θ,360)/359 - Encoded motor rotations into categorical integers.
3	Input values	Magnetic field magnitude and angle relative to the *x*-axis.
4	Targets	Stepper motor rotation, TME rotation, normalized *X*, *Y*, *Z* positions
5	Data splitting	Split data into 80% training and 20% testing, using stratified cross-validation for consistency.
6	ANN architecture	- Feedforward network with three hidden layers, each containing five neurons. - Activation: ReLU. - Training algorithm: Levenberg–Marquardt.
7	Output predictions	- Motor rotation (mapped to valid categories: 0°, 45°, 90°, 135°, 180°}). - TME rotation (mod (θ × 359), 360). - Positions (denormalized).
8	Implementation	MATLAB R2024b is used for neural network training and prediction

To further ensure model reliability, the possibility of overfitting was carefully examined by comparing training and testing errors. The close correspondence observed between the two datasets indicates that the network generalized effectively, avoiding overfitting to the training data. Additionally, performance metrics were evaluated across multiple cross-validation folds, further confirming that the ANN provided robust predictions rather than overfitting to a specific subset of data.

**Fig. 6:**
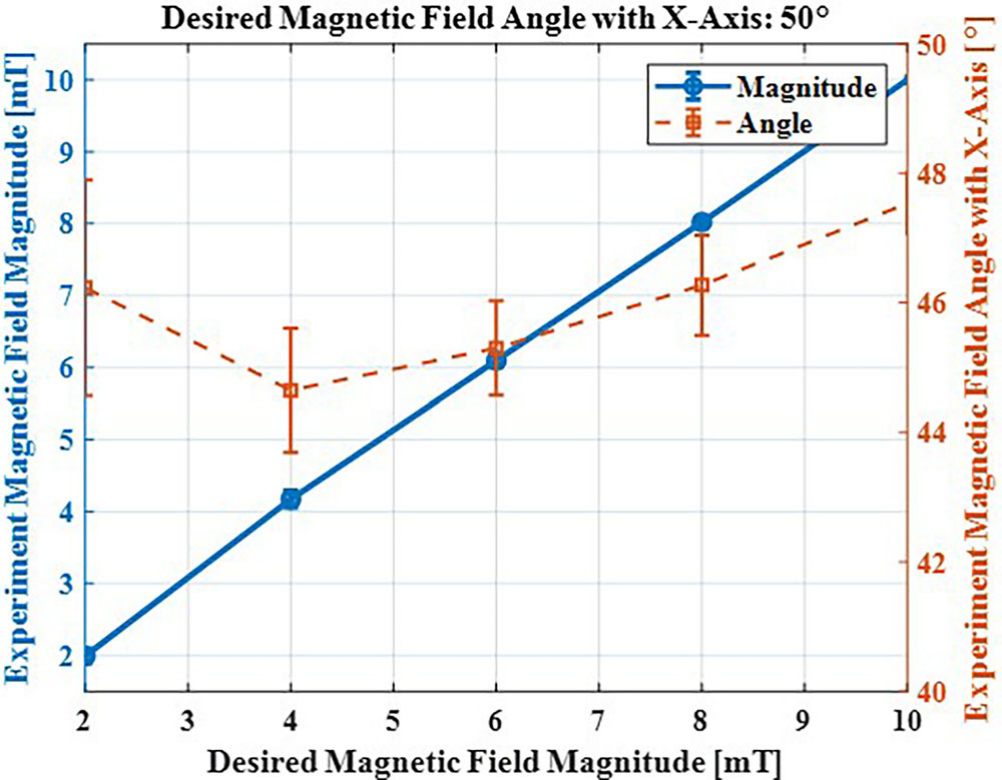
Comparison of experimental results with the desired magnetic field magnitude and angle relative to the *x*-axis (50°). Error bars indicate the maximum absolute error relative to the mean, calculated from three repeated experiments.

For validation, five input conditions were tested, with magnetic field magnitudes of [2, 4, 6, 8, 10 mT] and a field orientation angle relative to the *x*-axis fixed at 50°. Based on the algorithm’s outputs (robot positions, TME rotation, and stepper motor angles), 10 experimental trials were conducted for each condition. The results, shown in Fig. 6, indicate a maximum error of 4.4% for magnetic field magnitude and 10.7% for the angular orientation relative to the *x*-axis. This ANN framework successfully predicts and interpolates TME configurations based on input conditions, providing a robust foundation for system optimization and control.

### Different regions of the magnetic field

The results indicate that a TME can generate a wide range of magnetic field directions and strengths by adjusting three main parameters: (1) position, (2) motor rotation, and (3) end effector rotation. While it is possible to control the direction of the generated magnetic field within the workspace using a single TME, this control is limited to a specific region. It is not feasible to independently control the magnetic field direction across multiple regions of the workspace with only one TME.

However, by employing a dual TME configuration, both the magnetic field strength and direction can be effectively controlled across different regions within the workspace. Through the simulation platform, the concept of generating magnetic fields with varying directions across different regions of the workspace was demonstrated.

**Fig. 7:**
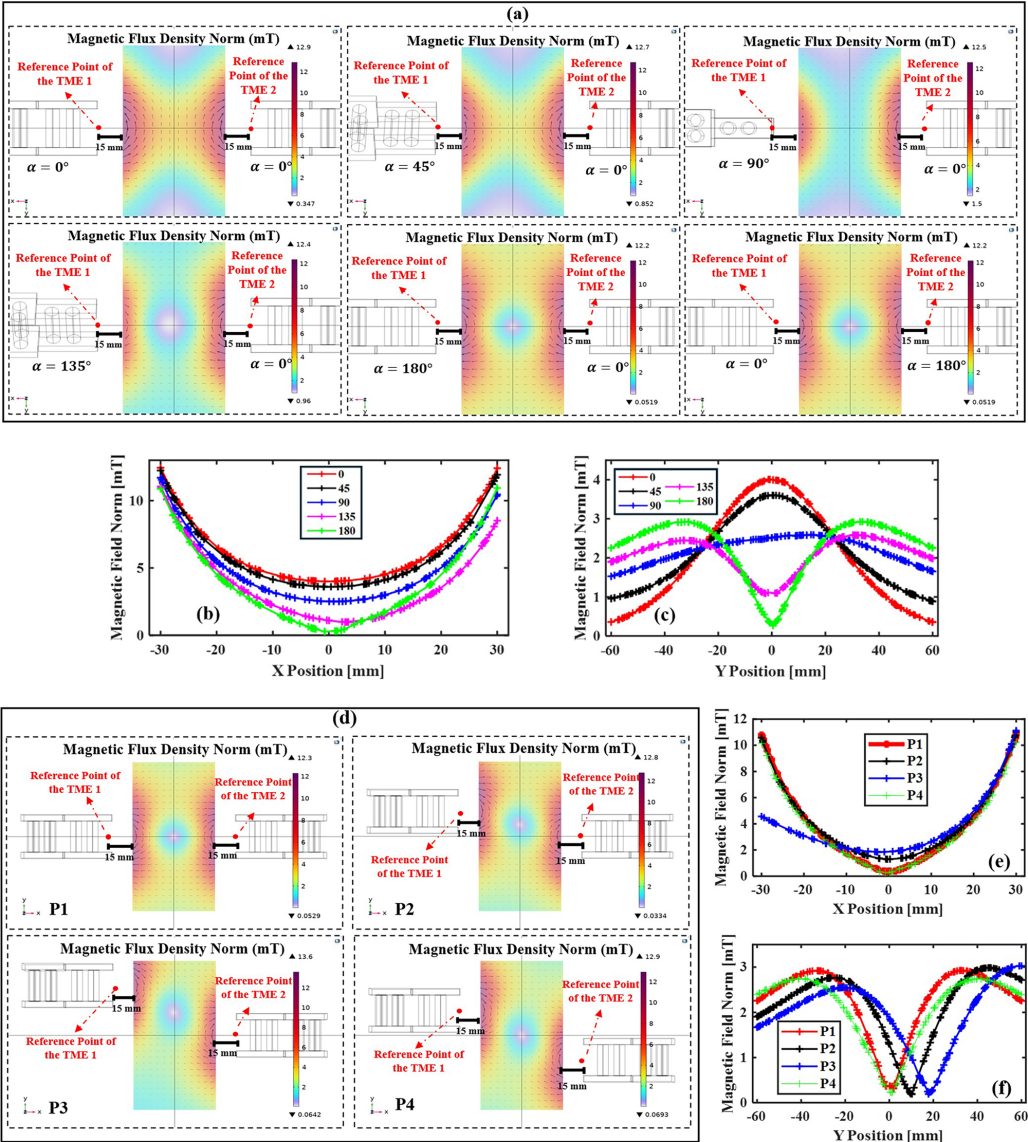
Effect of dual TME configurations on magnetic field distribution. **a** COMSOL simulation showing magnetic field regions generated in the workspace with dual TMEs (both ON) under different end effector rotations (EER). **b**, **c** Magnetic field norm along the *x*- and *y*-axes for rotation angles of 0°, 45°, 90°, 135°, and 180° with both TMEs fixed in the same position. **d** COMSOL simulations of magnetic field regions with dual TMEs at different reference point positions (P1–P4). **e**, **f** Magnetic field norm along the *x*- and *y*-axes for positions P1–P4, where reference points are set as: [(−45,0,0),(45,0,0)], [(−45,20,0),(45,0,0)], [(−45,40,0),(45,0,0)], and [(−45,20,0),(45,−20,0)].

As illustrated in Fig. 7, altering the end effector angle of the dual TME relative to one another affects both the direction and strength of the magnetic field across different regions of the workspace. However, the most dominant effect of end effector rotation is observed in the directional changes of the magnetic field (Fig. 7a–c). Furthermore, adjusting the relative positions of the TME reference points also influences the magnetic field’s direction and strength within the workspace. In this case, the most pronounced variations are observed in the magnetic field strength (Fig. 7d–f). Finally, another observation shows that modifying the motor rotation inside the TME structure, while keeping the magnetic field direction consistent across regions, leads to changes primarily in the field strength.

This configuration provides the capability for dynamic adjustment of the magnetic field direction and strength at multiple locations within the workspace. Furthermore, the dual TME system enables the generation of a field-free point (FFP), and by modifying the positioning and rotation of the TMEs, the location of the FFP can be precisely controlled.

The simulation was carried out under specific conditions; however, by analyzing the experimental dataset collected in the “Results” section, a detailed and comprehensive map of the magnetic field distribution generated by dual TMEs was constructed. This map characterizes both the direction and the strength of the magnetic field across the workspace. Additionally, the analysis allows for accurate prediction of the potential FFP locations, which is a critical factor for achieving precise control and optimization in magnetic manipulation.

## Discussion

Proof-of-concept studies confirm the validity of the study’s three main contributions: (1) the generation of a tunable magnetic field, (2) dual TMEs control with distinct magnetic actuation regions (multi-region control), and (3) enhanced field control using a data-driven approach for medical miniaturized robots’ applications.

In medical small-scale robotics, three main types of miniaturized robots are commonly utilized: untethered, tethered, and swarm-based systems. Table 2 provides an overview of this section, highlighting the efficiency of the proposed system and its contributions across various applications.

**Table 2:** Contributions of the proposed system across different miniaturized robotic applications

Type of robot		Contributions have been shown in the application
Untethered	Magnetic milli-carrier	Contribution 1: controlled speed adjustment of the magnetic carrier using a TME (Fig. 8c). Contribution 2: motion control (forward and backward) enabled by dual TMEs and magnetic region manipulation (Fig. 8d, e). Contribution 3: precise steering of the magnetic carrier through the desired junction by dynamically adjusting the magnetic TME’s conditions (position, rotation angle, and stepper motor angle) using an ANN (Fig. 8e).
Tethered	Fully soft continuum magnetic (FSCMs) robots	Contribution 1: controlled tip deformation using a TME for precise manipulation (Fig. 9d, e). Contribution 2: (a) enhanced shape and tip control of the soft robot through a dual TME for improved adaptability (Fig. 9f). (b) Shape formation capability in magnetic soft microrobots, with or without memory effect, achieved via magnetic region control (Fig. 9a, e). Contribution 3: shape forming of the magnetic soft robot by using data from [3], and an ANN has been developed.
Swarm	Magnetic nanoparticles (MNPs)	Contribution 1: controlled speed adjustment of the swarm MNPs using a TME (Fig. 10a). Contribution 3: precise steering of the magnetic carrier through the desired junction by dynamically adjusting the TME’s conditions (position, rotation angle, and stepper motor angle) using an ANN (Fig. 10d).

### Untethered milli-robot actuation

**Fig. 8:**
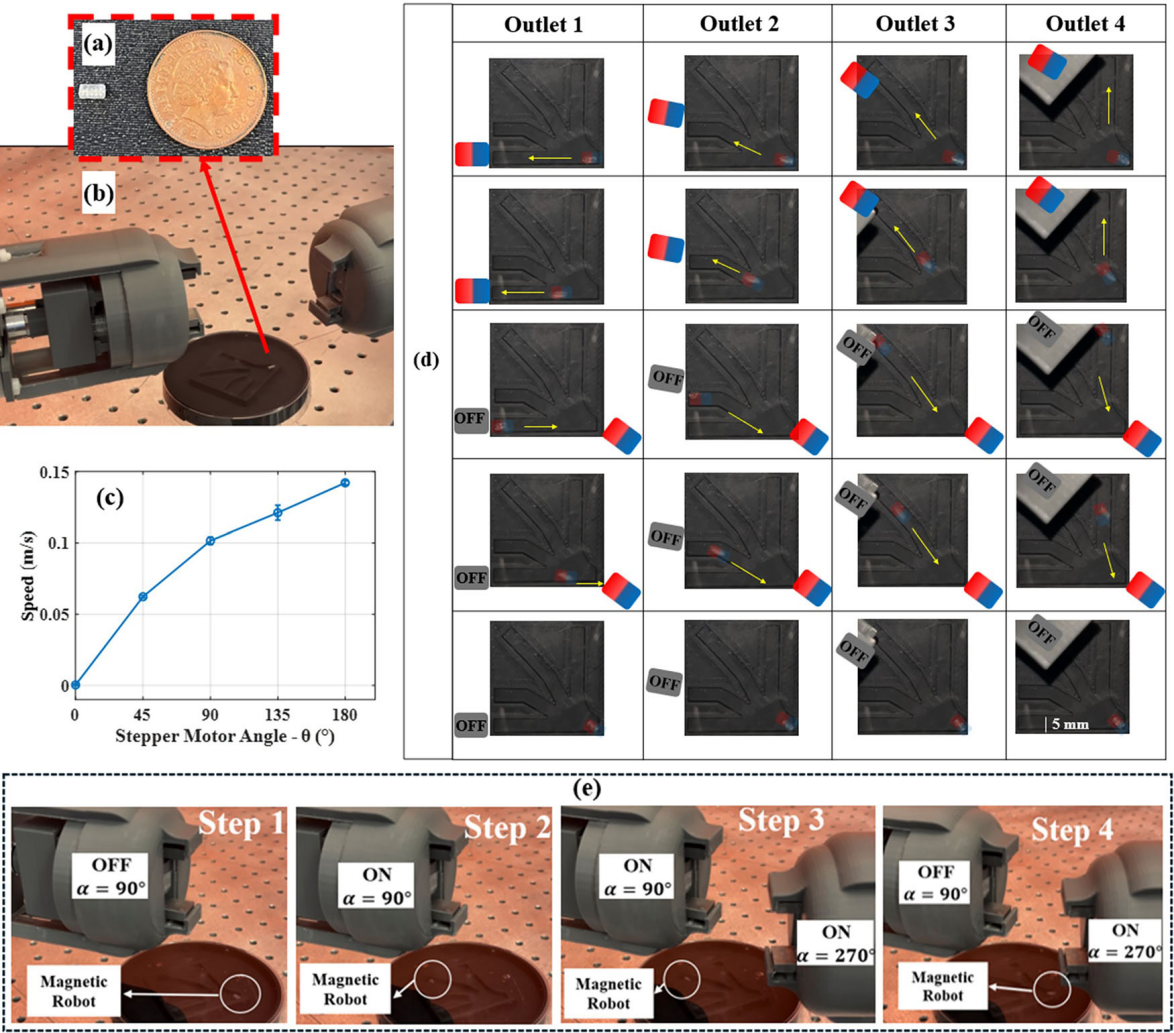
TME for steering the magnetic milli-carrier. **a** Fabricated millimeter-scale magnetic carrier dimensions compared to a 1-penny coin. **b** The designed phantom and experimental setup consist of dual TME. **c** Effect of stepper motor rotation degree (θ) on the average speed of the milli-magnetic carrier (number of tries = 3, and error bars indicating the maximum absolute error relative to the mean). **d** Sequential movements of the magnetic carrier. Each photo includes an overlay highlighting the position and magnetic orientation of the carrier. **e** Magnetic field directions in different regions are controlled using TME.

To assess the performance of the developed TME in controlling magnetic robots, a cylindrical magnetic milli-carrier was designed with a radius of 1.5 mm and a length of 4 mm. This milli-carrier features two cylindrical neodymium-50 magnet cores of identical dimensions (1 mm radius and 2 mm length) embedded within its structure (Fig. 8a) as a demonstrative magnetic robot.

A phantom model comprising four distinct paths (0°, 30°, 60°, and 90°) was constructed to evaluate the performance of the proposed TME and the ANN model (Fig. 8b). Additionally, Fig. 8c illustrates the impact of the stepper motor’s rotation on the average speed of the milli-magnetic carrier. The average speed is determined from the displacement of the center of mass of the magnetic milli-carrier during a defined time interval. In this experiment, the TME was fixed at a predetermined position, with the distances between the center of the magnetic carrier and the reference point along the *x*, *y*, and *z* axes set to 35 mm, 20 mm, and 0 mm, respectively. The TME was oriented at 270°. The motor’s rotation angle varied from 180° to 0° in 45° increments. The results indicate that adjusting the motor’s angle alters the generated magnetic field’s magnitude, which directly influences the average speed of the milli-magnetic carrier.

Based on the experiment results in Fig. 8c, the generated magnetic field on the carrier in the ON condition was analyzed. The calculated magnetic field strength and direction (determined by different junction angles in the designed phantom) were provided to the ANN to identify the optimal TME condition for generating the required magnetic field in the desired direction. Additionally, the minimum magnetic field magnitude needed to initiate the motion of the milli-magnetic carrier was determined, along with the field direction required for precise steering. Using these two inputs (strength and direction of the magnetic field), the ANN calculated the positioning and variable values of the TME for successful navigation. The milli-robot’s movement along predefined pathways was controlled by dual TME (Fig. 8b).

Figure 8d illustrates the milli-robot’s trajectory through the phantom, guided by the outputs of the ANN for TME condition settings. In this proof-of-concept experiment, by modulating the magnetic field values, the motion of the milli-carrier was effectively controlled within the workspace using the dual-TME setup (shown in Fig. 8d). In this scenario, the dual TME units operated independently. One TME facilitated forward movement (α = 90°), while the second enabled reverse motion (α = 270°). This dual-TME configuration improved adaptability to diverse operational scenarios.

Furthermore, COMSOL simulations were conducted to estimate the initial magnetic field and force required to initiate the motion of the magnetic milli-carrier, which was determined to be approximately 1.8 mT. An ANN was employed to determine the optimal positioning of the TMEs to guide the milli-carrier along the desired trajectory toward the target point. The schematic illustrating the relative positioning of the TME with respect to the experimental phantom for the first outlet configuration is presented in Fig. S1 of the Supplementary Materials. Figure S2 (Supplementary Materials) further depicts the magnetic field distributions under both ON and OFF conditions across the entire experimental phantom.

According to the TME positioning, the magnetic field within the workspace of the milli-carrier remains below 1 mT when the TME is in the OFF state (Fig. S2b) in the Supplementary Materials. Consequently, this field strength is insufficient to generate a magnetic force capable of initiating motion in the milli-carrier. In contrast, when the TME is activated (ON condition), the magnetic flux density norm at the center of mass of the magnetic milli-carrier reaches approximately 1.73 mT, with magnetic field components (${B}_{x}$, ${B}_{y}$, ${B}_{z}$) = (−1.60 mT, −0.66 mT, 0 mT). Under the OFF condition, the magnetic field has a negligible influence on the milli-carrier’s motion throughout its workspace, with the field norm at the starting position measured at approximately 0.15 mT.

The range of magnetic forces generated for the milli magnetic carrier across all four outlet configurations is presented in Fig. S3 (Supplementary Materials).

The sequential movements of the magnetic carrier (Fig. 8d) are guided by TMEs. Yellow arrows indicate the direction of motion of the magnetic milli-robot in Fig. 8d. To enhance clarity, a symbolic representation of the magnets is overlaid on the image, clearly distinguishing the north and south poles of both the TMEs and the magnetic carrier. The Supplementary Video S3 demonstrates how the calculated magnetic field, generated through an ANN-based approach, navigates the robot’s motion in various directions. It also shows how the tunable (ON–OFF) function transitions control between the TMEs, enabling smooth magnetic field direction in the specific region switching. The different magnetic field regions generated by switching the dual TME between ON and OFF states at a fixed position for the second, third, and fourth outlets are shown in Figs. S4, S5, and S6 of the Supplementary Material.

Figure 8e illustrates how different magnetic field directions through the workspace can influence the movement of the magnetic carrier through a tunable magnetic field. As shown in Video S4 of the Supplementary Videos, a COMSOL simulation was also performed to clearly visualize the changes in the magnetic field regions generated by the dual TME. In the experiment (demonstrated in the Supplementary Video S4), the magnetic carrier is initially attracted by the first TME (Step 2 in Fig. 8e). Once the carrier reaches the desired position, the second TME moves into its predefined configuration. However, since the second TME’s control field direction is inactive at this stage, it does not exert any influence on the carrier (Step 3 in Fig. 8e). When the magnetic field of the first TME is turned OFF, the carrier comes under the influence of the second TME and moves accordingly in the predefined direction (Step 4 in Fig. 8e). For enhanced control of magnetic milli-carriers and untethered magnetic robots, real-time feedback is essential for maintaining a connection with the controller. Based on the updated location, average speed, and acceleration at each time step, the new position of the TME can be calculated using the outputs of the ANN.

In this study, an open-loop control approach was implemented to steer the magnetic milli-carrier, where an ANN was used to determine the TME conditions required to generate the desired magnetic field for simple trajectory guidance. While this method demonstrated the feasibility of data-driven magnetic field generation, precise navigation along complex 3D paths or in vivo scenarios will require a more advanced dynamic model and closed-loop feedback control integrated with the ANN for real-time magnetic field modulation.

### Tethered miniaturized robot actuation

Tethered robots, such as magnetic soft continuum robots, are among the most promising magnetic robots for minimally invasive medical interventions. They offer the possibility for both tip deflection^38^ or shape control^3,39–41^, depending on design. One of the most critical factors in the shape formation of magnetic soft materials is the memory effect. The memory effect refers to the phenomenon where the final shape of a magnetic soft robot is influenced not only by the external magnetic field but also by its initial shape. This introduces an additional constraint: when a PM is used for shape formation, the path taken by the magnet during movement may affect the resulting shape of the magnetic soft microrobot^26^.

**Fig. 9:**
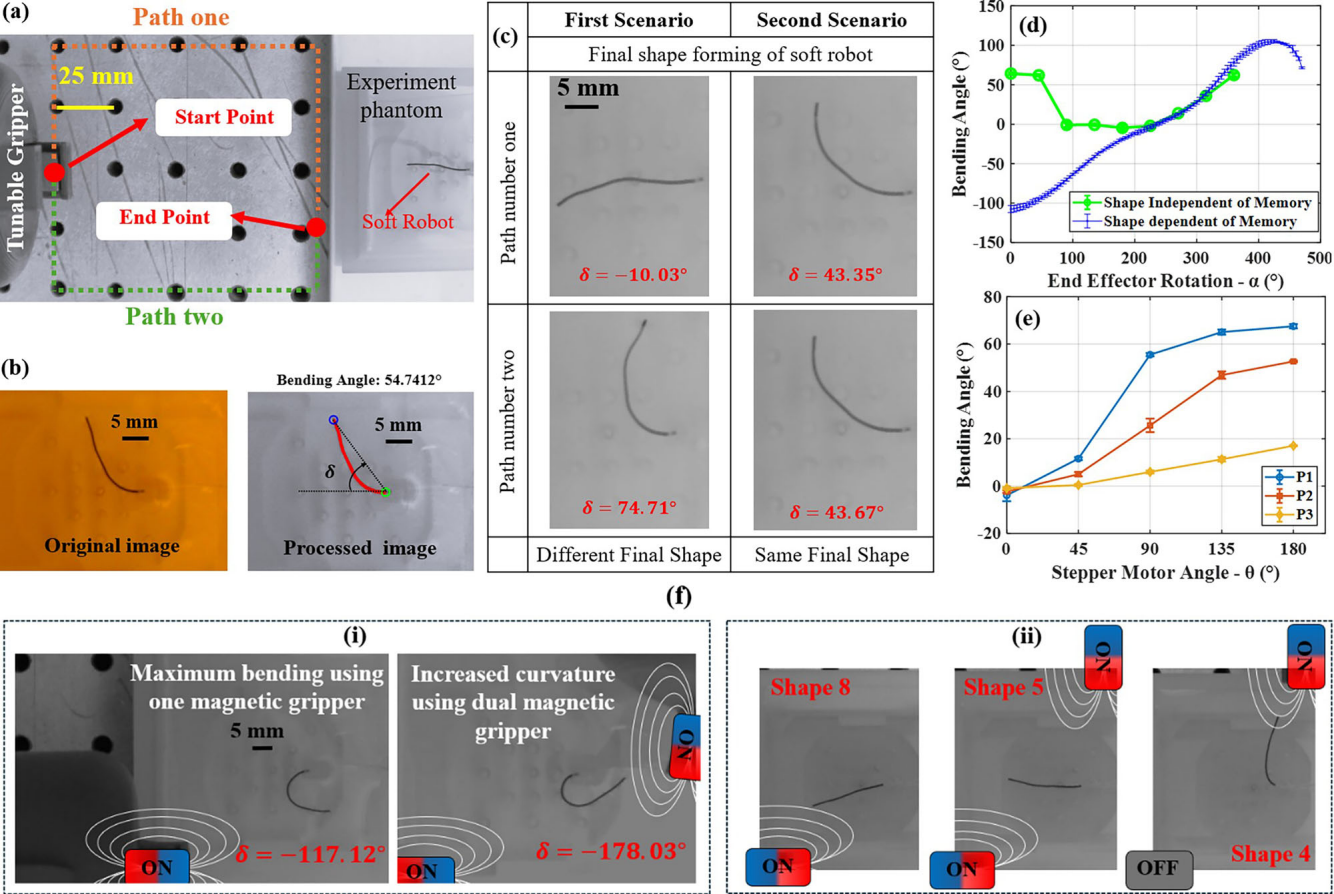
Tip deformation and shape formation of the magnetic soft microrobot under the influence of TME. **a** Experimental setup for the untethered application test. **b** A comparison between the original and processed image. **c** The shape formation of the magnetic soft robot in two scenarios is illustrated. The reference point for the start and end point is positioned at a distance of (130 mm, 15 mm, 0 mm) and (30 mm, 15 mm, 15 mm) from the tip of the magnetic soft robot, respectively. **d** The effect of TME rotation on the bending angle of the magnetic soft robot, comparing results with and without the memory effect (number of tries = 3, and error bars indicating the maximum absolute error relative to the mean). The reference point is positioned at a distance of (30 mm, 15 mm, 15 mm) from the tip of the magnetic soft robot. **e** The effect of the stepper motor angle on the bending angle of the magnetic soft robot when the reference point is located at three different distances (P1, P2, and P3) from its tip. The positions of P1, P2, and P3 are (35 mm, 25 mm, 25 mm), (40 mm, 25 mm, 25 mm), and (45 mm, 25 mm, 25 mm), respectively (number of tries = 3, and error bars indicating the maximum absolute error relative to the mean). **f** Magnetic field direction control using a dual TME: (i) expansion of the magnetic soft robot’s bending motion using a dual TME (Supplementary Video S6) and (ii) formation of different shapes using a dual TME.

To investigate the memory effect on the shape formation of a magnetic soft robot, a fully soft continuum magnetic (FSCMs) microrobot with a length of 22 mm and a diameter of 600 μm (similar to the soft robot studied in ref. ^3^) was used. Two different movement paths for the TME were considered (Fig. 9a).

Additionally, the bending angle (δ) was defined as a parameter to assess the effect of the external magnetic field on the tip deformation of the magnetic soft robot (Fig. 9b). To calculate the bending angle, an image processing code was developed in MATLAB. This code detects the boundaries of the soft robot and fits a curve to its shape. In Fig. 9b, the red line represents the detected boundaries obtained through image processing, while the blue and green circles indicate the detected tip and end of the soft robot, respectively.

In continuation of this study, two experimental scenarios were considered:

Scenario 1: the TME moves from the start point to the end point along two predetermined paths while remaining in the ON condition.

Scenario 2: the TME moves from the start point to the end point along the same two predetermined paths while in the OFF condition. Upon reaching the end point, the stepper motor rotates, switching the TME to the ON condition.

The videos demonstrating the shape formation of the magnetic soft robot in these two scenarios are presented in Supplementary Video S5.

The final shape formation of the magnetic soft robot in each scenario is shown in Fig. 9c. As the results indicate, when the TME is ON, the movement path taken to reach the endpoint significantly influences the robot’s final shape. However, when the TME is OFF, the shape of the magnetic soft robot remains unchanged, regardless of the selected path. Notably, upon reaching the endpoint and switching the TME to ON, the magnetic soft robot assumes a unique shape.

The proposed TME has the capability of shaping the soft robot both with and without the influence of the memory effect. Figure 9d illustrates the influence of TME rotation on the tip bending angle of the magnetic soft robot. As the results demonstrate, by adjusting the TME angle from 180° in the ON state, we can dynamically increase or decrease the bending angle of the soft robot in a smooth and continuous manner. However, without utilizing the memory effect, the tip bending behavior becomes inconsistent and discontinuous. Furthermore, by varying the magnetic field strength through adjustments to the motor angle, the bending angle of the magnetic soft robot can be precisely controlled, as shown in Fig. 9e.

The effectiveness of the dual TME in expanding the bending angle and enabling the creation of a wide range of shape formations is demonstrated in Fig. 9f. Using the data from the ref. ^3^, the external magnetic field required for shape formation of the magnetic soft robot was determined, and the best optimal configuration to generate this via the TME obtained by the ANN. Figure 9f(ii) shows that, by utilizing the dual TME and controlling the magnetic field directions, we can achieve a wide range of shapes on the magnetic soft robot. Through the OFF and ON capabilities of the TME, as well as stepper motor rotation, we have precise control over the bending angle of the magnetic soft robot.

Moreover, a similar experiment was conducted on soft robots with a larger diameter (2 mm). The results show that the tip deformation and overall shape formation of the magnetic soft microrobot are also influenced by the memory effect. This indicates that when applying the TME to larger magnetic soft microrobots, more precise control over their shape and tip configuration can be achieved. Such capabilities highlight the potential use of these robots in medical applications, including endoscopy and colonoscopy.

### Swarm MNPs

**Fig. 10:**
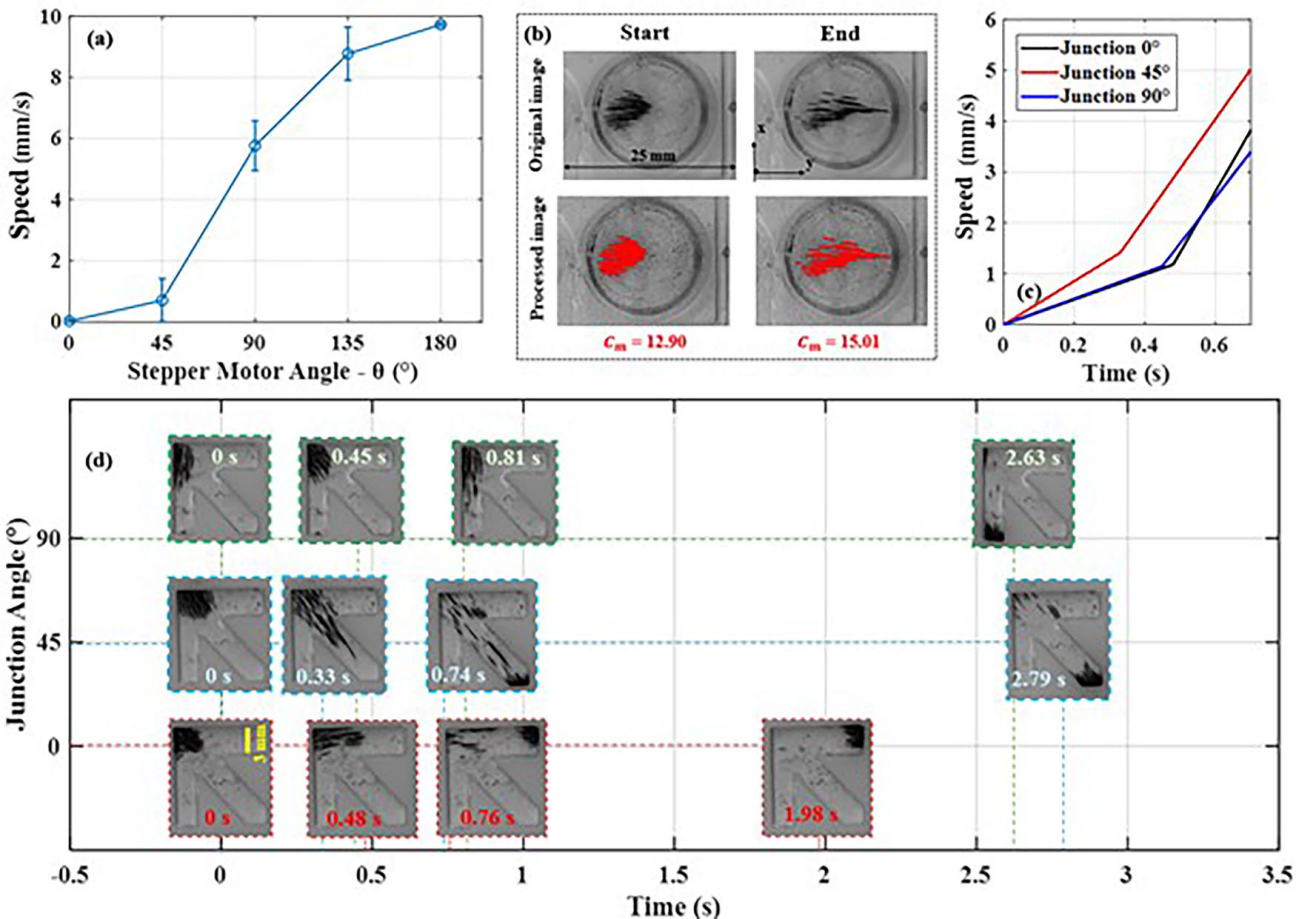
Directional control of swarm MNPs using a TME. **a** Effect of the TME at a fixed point on the speed steering of the swarm of MNPs. The reference point of the TME is fixed at varying distances from the center of the cylindrical glass (15 mm, 0 mm, and 20 mm). Error bars indicate the maximum absolute error relative to the mean, calculated from three repeated experiments. **b** Processed data through image processing. ${C}_{m}$ represents the center of mass of the MNPs, calculated as $\sqrt{{{x}_{m}}^{2}+{{y}_{m}}^{2}}$, where ${x}_{m}$ and ${y}_{m}$ are the *x* and *y* coordinates of the center of mass of the MNPs. **c** Speed changes of the swarm of MNPs at three junctions based on the output of the ANN. **d** Motion of the swarm of MNPs through different junctions over time.

To investigate the magnetic field direction control in different regions of the workspace using the TME, Iron (II, III) oxide powder with particle sizes ranging from 50 to 100 nm was utilized. A solution was prepared by mixing 4000 µL of water with 0.01 g of MNPs, resulting in a concentration of 2.5 mg/mL. To examine the impact of the TME, 1 mL of the solution was added to a cylindrical glass with an inner diameter of 15 mm. In this study, the TME was fixed in position, and the average speed of the swarm of MNPs was observed under varying magnetic strengths, which were adjusted by altering the stepper motor angle (Fig. 10a). Image processing techniques in MATLAB were used to analyze the collected data (Fig. 10b).

As demonstrated in Fig. 10a, the TME exerts a significant influence on regulating the speed and trajectory of the swarm of magnetic nanoparticles (MNPs). To investigate this effect in greater detail, a phantom model incorporating three junctions at angles of 0°, 45°, and 90° was subsequently designed (Fig. 10a).

Using data shown in Fig. 10a, which indicates an average speed of 5 mm/s, the essential magnetic field has been calculated. Based on the junction angle, the magnetic field strength and direction have been provided as input to the ANN. By analyzing the output from the ANN, the optimal position and conditions for the TME to generate the necessary magnetic field were determined. The results of this experiment are presented in Fig. 10b, c. The movement of magnetic nanoparticles through each junction, achieved by adjusting the TME with ANN, is demonstrated in Supplementary Video S7. Based on the output of the ANN, the TME’s ability to steer the swarm of MNPs was determined. The average speeds from 0 to 0.7 ms for the junctions at 0°, 45°, and 90° were 4.54 mm/s, 5.42 mm/s, and 4.38 mm/s, respectively. The average deviation from the desired average speed of 5 mm/s was 4.4%.

## Conclusion

This study presents a TME. The proposed system enables precise modulation of the magnetic field strength and direction without requiring significant mechanical movement (stationary field control). The computational simulations, validated by experimental data, demonstrate the capability of the TME to switch between ‘ON’ and ‘OFF’ magnetic field states. Moreover, the material and size variations of the PMs within the system were analyzed, revealing that while magnet size has a substantial impact on the generated field, the material selection can be adjusted based on specific application needs.

In addition to the TME design, the incorporation of an ANN enhances system adaptability by predicting and optimizing TME configurations. This capability ensures precise control of magnetic fields and the reliable navigation of a diverse range of magnetic agents, including untethered carriers, tethered miniaturized robots, and MNP swarms, as demonstrated in validation experiments. The dual-TME configuration further expands the workspace and enhances motion control by introducing distinct regions of control.

Experimental proof-of-concept investigations confirmed the TME’s ability to manipulate magnetic fields remotely, offering potential for applications in delicate medical procedures such as microsurgery, drug delivery, and minimally invasive robotic systems.

Beyond the proof-of-concept demonstrations, the dual-TME configuration enables multi-region magnetic control and the generation of field FFPs, supporting manipulation of multiple agents. However, the system has been tested mainly under controlled lab conditions, and real-time feedback in dynamic environments remains limited. ANN-based control may face challenges under unpredictable disturbances.

Future work should explore scalability to larger or more complex workspaces, integration of real-time sensing, validation in physiologically relevant environments, and enhanced ANN frameworks for adaptive and robust control.

Overall, this study establishes a foundation for tunable magnetic field manipulation with single and dual TME configurations, with potential for precise and safe actuation in medical microrobotics.

## Methods

### Design and mechanism of a tunable end effector

**Fig. 11:**
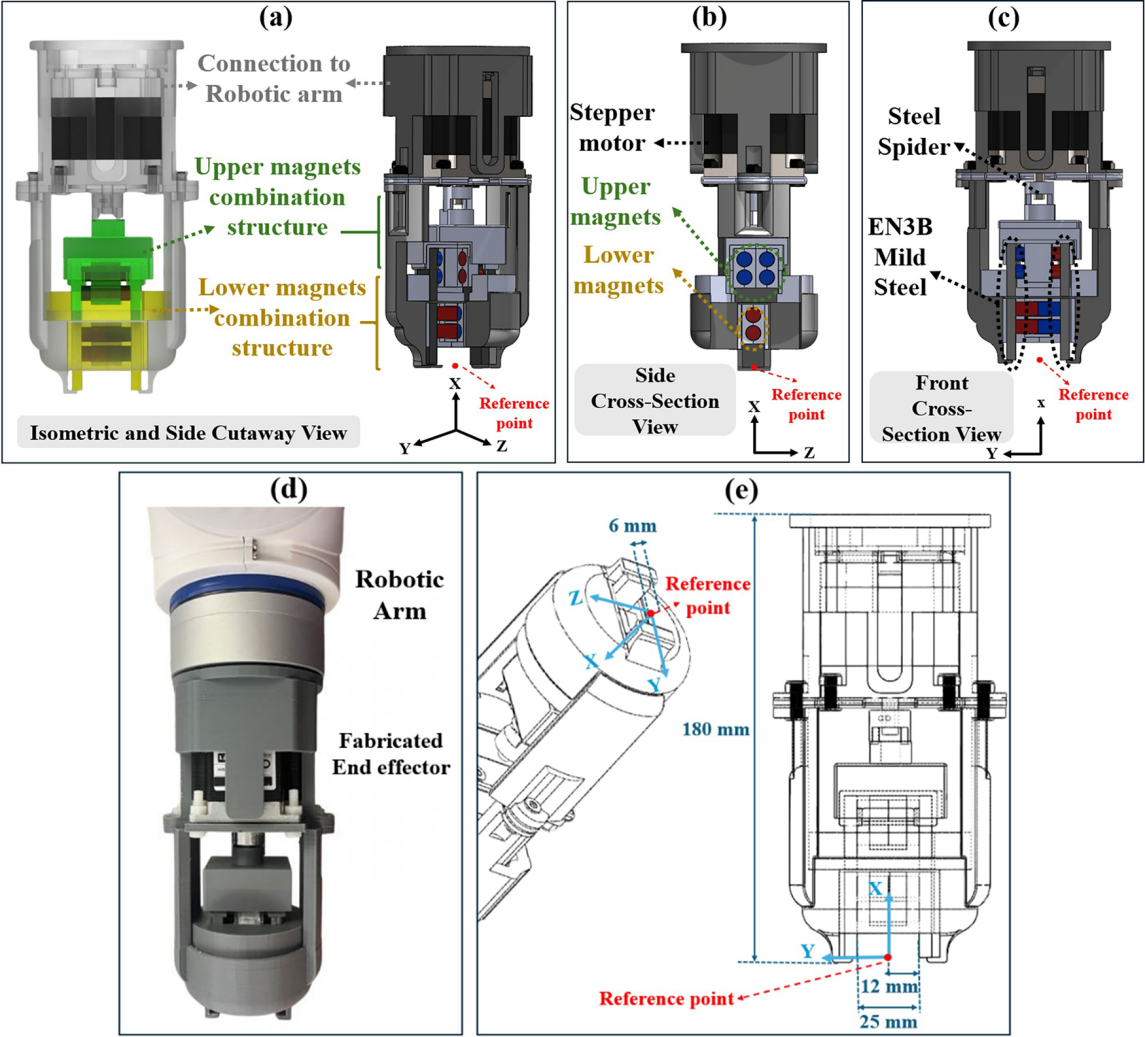
Design and fabrication of the TME. Axes (*x*, *y*, *z*) are included for orientation. **a**–**c**, **e** SOLIDWORKS (Version 2024) models: **a** Isometric and side cutaway view showing the robotic arm connection and the upper (green) and lower (yellow) magnet assemblies. **b** Side cross-sectional view of the magnet arrays and stepper motor configuration. **c** Front cross-sectional view highlighting structural supports, including the steel spider and EN3B components. **d** Fabricated TME assembled for experimental use. **e** Dimensional schematic of the final TME; the red dot marks the reference point (**r**_**p**_) used for data collection.

To design a PM-based system with a tunable magnetic field, it is essential to configure the PMs within the end effector such that their relative orientations enable internal cancellation or external direction of the magnetic field. This configuration facilitates ON/OFF switching and variable magnetic field effects. The terms ON and OFF refer to the operating configurations of the TME. In the ON configuration, the TME produces a magnetic field strong enough to drive the motion of the magnetic object, whereas in the OFF configuration, the arrangement of the PMs results in a field too weak to initiate motion. To deliver this concept, the TME is designed with three key components: (1) the upper assembly, (2) the lower assembly, and (3) the connection assembly, as illustrated in Fig. 11.

The upper assembly comprises a stepper motor integrated with an upper magnet structure. This structure includes a steel spider coupling the stepper motor to the magnet assembly. The magnet assembly consists of two cubical EN3B mild steel components, each measuring 24 mm × 30 mm × 5 mm. Four cylindrical N42 Neodymium magnets, each with dimensions of 8 mm in diameter and 25 mm in length, and a pulling force of 3.1 kg, are positioned in a uniform orientation within a custom-designed 3D-printed frame. Together, these components form the complete upper magnet structure.

The lower assembly contains two EN3B mild steel components, each with dimensions of 35 mm × 13 mm × 5 mm, along with two additional N42 Neodymium magnets identical to those used in the upper assembly. These magnets are also oriented in the same direction and housed within a similar 3D-printed structure. The magnets in both the upper and lower assemblies are aligned with their north-south poles oriented in the same direction to ensure uniform magnetic interaction across the structure. The steel parts guide and concentrate the magnetic flux generated by the PMs.

The connection assembly consists of 3D-printed components specifically designed to securely attach both the upper and lower assemblies to the robotic arm. In this configuration, the magnets in the upper and lower assemblies are aligned in the same direction. However, their relative orientation can be adjusted to produce a variable magnetic field effect.

In the ON state, the upper and lower assemblies are oriented at a 180° offset. This alignment maximizes the combined effect of the six magnets, generating a strong and focused magnetic field. At a 0° offset, the magnets are oriented to oppose each other, effectively canceling the net magnetic field and establishing the OFF condition with minimal magnetic influence.

### Computational modeling

The magnetic field produced by a cylindrical magnet can be calculated using the magnetic scalar potential ɸ derived from the magnetization M. For a uniformly magnetized body, the magnetic scalar potential outside the magnet is given by ref. ^42^:

1 $$\Phi ({{{\rm{r}}}}_{{{\rm{p}}}})=\frac{1}{4{{\rm{\pi }}}}\int\limits_{{{\rm{V}}}}\frac{\nabla .{{\rm{M}}}({{{\rm{r}}}}_{{{\rm{c}}}})}{|{{{\rm{r}}}}_{{{\rm{p}}}}-{{{\rm{r}}}}_{{{\rm{c}}}}|}{{\rm{d}}}{{{\rm{V}}}}^{{\prime} }$$

where ${{\mbox{r}}}_{{\mbox{p}}}$ represents the position vector of the point at which the field is being evaluated, ${{\mbox{r}}}_{{\mbox{c}}}$ demonstrates the position vector inside the magnet, and *V* is the volume of the magnet. ${{\rm{d}}}{{{\rm{V}}}}^{{\prime} }$ is the differential volume element used in the integral over the volume of the magnet.

For a cylindrical magnet, the magnetic field can be expressed as the gradient of the scalar potential^43^:

2 $${{\rm{B}}}({{{\rm{r}}}}_{{{\rm{p}}}})=-{{{\rm{\mu }}}}_{0}\nabla \Phi ({{{\rm{r}}}}_{{{\rm{p}}}})$$

where ${\mu }_{0}$ is the permeability of free space, equal to $4{{\rm{\pi }}}\times 1{0}^{-7}{{\rm{T}}}.{{\rm{m}}}/{{\rm{A}}}\,$.

The magnetic field in cylindrical coordinates can be decomposed into three components: the radial field, axial field, and azimuthal magnetic field. Assuming the magnet is a uniformly magnetized cylindrical magnet, the azimuthal magnetic field $({{\mbox{B}}}_{{{\boldsymbol{\theta }}}})$ component is zero due to the symmetry of the system. The radial magnetic field (${{\mbox{B}}}_{{{\boldsymbol{r}}}}$) and axial components of the magnetic field (${{\mbox{B}}}_{{{\bf{z}}}}$) can be modeled as follows:

3 $${{{\rm{B}}}}_{{{\bf{r}}}}({{\mbox{r}}}_{{{\boldsymbol{p}}}},{{{\bf{z}}}}_{{{\boldsymbol{p}}}})=\frac{{\mu }_{0}{\mu }_{{{\boldsymbol{r}}}}}{{{\boldsymbol{2}}}}{\int }_{\!\!\!\!-\frac{{{\boldsymbol{h}}}}{{{\boldsymbol{2}}}}}^{\frac{{{\boldsymbol{h}}}}{{{\boldsymbol{2}}}}}\frac{({{{\bf{z}}}}_{{{\boldsymbol{p}}}}-{{{\bf{z}}}}_{{{\boldsymbol{c}}}})}{({{{\mbox{r}}}_{{{\boldsymbol{p}}}}}^{{{\boldsymbol{2}}}}+{{({{{\bf{z}}}}_{{{\boldsymbol{p}}}}-{{{\bf{z}}}}_{{{\boldsymbol{c}}}})}^{{{\boldsymbol{2}}}}})^{\frac{{{\boldsymbol{3}}}}{{\boldsymbol{2}}}}}{{\boldsymbol{d}}}{{{\bf{z}}}}_{{{\boldsymbol{c}}}}$$

And the axial field for when ${{\mbox{r}}}_{p}$ is equal to zero can be described in ref. ^44^

4 $${{{\rm{B}}}}_{{{\bf{z}}}}\left({{{\bf{z}}}}_{{{\bf{p}}}}\right)=\frac{{\mu }_{0}{\mbox{M}}}{{{\boldsymbol{2}}}{{{\bf{z}}}}_{{{\bf{p}}}}}\left(\frac{{{{\bf{z}}}}_{{{\bf{p}}}}+\frac{{{\boldsymbol{h}}}}{{{\boldsymbol{2}}}}}{\sqrt{{\left(\frac{{{\boldsymbol{d}}}}{{{\boldsymbol{2}}}}\right)}^{{{\boldsymbol{2}}}}+{\left({{{\bf{z}}}}_{{{\bf{p}}}}+\frac{{{\boldsymbol{h}}}}{{{\boldsymbol{2}}}}\right)}^{{{\boldsymbol{2}}}}}}-\frac{{{{\bf{z}}}}_{{{\bf{p}}}}-\frac{{{\boldsymbol{h}}}}{{{\boldsymbol{2}}}}}{\sqrt{{\left(\frac{{{\boldsymbol{d}}}}{{{\boldsymbol{2}}}}\right)}^{{{\boldsymbol{2}}}}-{\left({{{\bf{z}}}}_{{{\bf{p}}}}+\frac{{{\boldsymbol{h}}}}{{{\boldsymbol{2}}}}\right)}^{{{\boldsymbol{2}}}}}}\right)$$

where *d* and *h* are the diameter and height of the cylindrical magnet. M represents the uniform magnetization of the cylindrical magnet, and ${\mu }_{{{\boldsymbol{r}}}}$ is the relative permeability of the PM.

As illustrated by Eqs. 3 and 4, the generated field depends on the magnet’s geometry and material properties. Analytical solutions for cylindrical magnets require solving complex integrals, which can pose challenges for systems with multiple magnets or arbitrary orientations, as seen in our design. Therefore, in this study, a finite element analysis (FEA) based method is used.

**Fig. 12:**
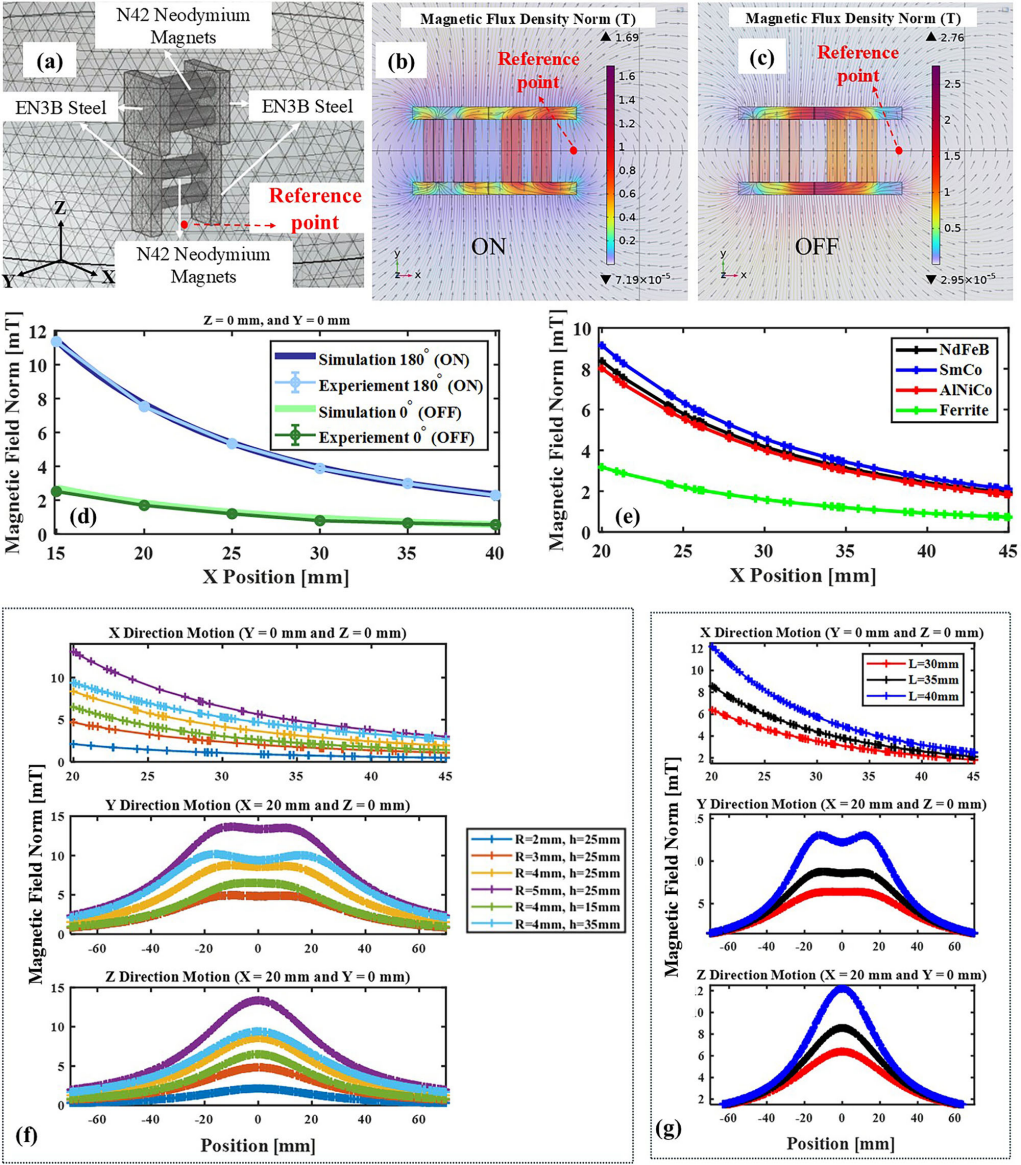
Simulation of the TME in COMSOL. **a** Geometry and mesh setup. **b** Magnetic field in “ON” condition. **c** Magnetic field in “OFF” condition with streamlines and color map representing the magnetic flux density norm. Gray vectors illustrate the magnetic flux in both (**b**, **c**), with the vectors normalized for clarity. **d** Comparison of simulated and experimental magnetic field variations. Error bars indicate the maximum absolute error relative to the mean, calculated from three repeated experiments. **e** Magnetic field norm along the *x*-axis for different materials (*y* and *z* = 0) in ON state. **f** Magnetic field norm vs magnet size (*R* = radius, *h* = height). **g** Magnetic field norm vs lower iron size (*L*).

To analyze the magnetic behavior of the TME, a COMSOL Multiphysics® 6.0 software package was used. The model focused on simulating the magnetic fields generated by the six cylindrical PMs embedded between EN3B mild steel parts (Fig. 12a).

The physics interface for magnetostatics was used to model the PMs and their interaction within the assembly. The mesh optimization’s size was employed to ensure convergence and accuracy, particularly in regions of high magnetic flux density around the magnets. The boundary conditions were defined to simulate the generated magnetic field in different scenarios.

Figure 12b illustrates the ON position, where the six magnets generate a strong outward magnetic flux. In contrast, Fig. 12c shows the OFF position, achieved by rotating the upper assembly to a secondary orientation, resulting in opposing magnetic fields that largely cancel each other. Additionally, Fig. 12c reveals a strong internal magnetic field generated between the upper and lower assemblies.

The COMSOL simulation was validated by comparing its results with experimental data under identical conditions. To assess field symmetry, magnetic fields in the ON and OFF positions were measured along two cut lines with the same *z*-values and *y*-values. Figure 12d shows the field variation in the *X*-direction. The error between simulated and experimental results was consistently below 10%, showing a strong correlation. The differences between simulation and experimental data likely stem from material imperfections or slight magnet mispositioning.

Fabricating TME with varying sizes and materials of PMs can be a time-intensive process. Therefore, the computational model serves as a tool to investigate the influence of structural modifications in the TME design. This approach aids in determining the optimal material and size of the magnets based on the intended application and workspace.

In this study, particular attention was given to evaluating the efficiency of the TME for specific in-vitro applications. To this end, the workspace dimensions, the distance between the workspace and the TME reference point, and the maximum active magnetic field strength within the workspace were analyzed. Based on these criteria, a magnetic field of up to 12 mT at a distance of 15 mm from the reference point was identified as sufficient for the intended proof-of-concept experiments. Using COMSOL simulations, the most compact configuration capable of generating this field strength was designed and subsequently fabricated.

It is important to note that this design does not represent a limitation. For alternative applications, higher magnetic fields at greater distances can be achieved by modifying the dimensions of the PMs within the TME structure, as verified by simulation studies. As demonstrated in the supplementary materials, adjusting the internal structural dimensions of the TME can produce magnetic fields of up to 25 mT and 45 mT at a distance of 15 mm (Table S3 and Fig. S7 in the Supplementary Materials). Furthermore, if the goal is to maintain a magnetic field of 12 mT at larger distances, optimized design parameters can be determined through simulation to guide TME design and fabrication (Table S4 and Fig. S8 in the Supplementary Materials).

The following section explores three key structural parameters in the design of the TME: (1) the material of the embedded PMs, (2) the size of the PMs, and (3) the size of the steel component embedded within the TME.

To examine the effect of magnet material in the TME, four types were tested: Neodymium Iron Boron (NdFeB), Samarium Cobalt (SmCo), AlNiCo, and Ferrite. NdFeB generates strong fields, SmCo offers temperature stability and corrosion resistance, AlNiCo is thermally stable, and Ferrite is cost-effective with good corrosion resistance. Figure 12e shows that variations in magnet material have a minor impact on the magnetic field under the ON condition of TME. Therefore, material selection can be adjusted based on specific application requirements.

As the cylindrical PMs are embedded within the TME, their size significantly influences the generated magnetic field. Two key volume parameters were examined: radius and length. The results of this investigation are presented in Fig. 12f. The data demonstrate that, for magnets of the same length, an increase in radius results in a proportional increase in the magnitude of the magnetic field in all directions. Conversely, for magnets with the same radius but varying lengths, an increase in length leads to a significant rise in the magnetic field magnitude only along the *X*-direction (aligned with the magnet’s length), while no noticeable changes are observed in the other directions.

This study evaluated three sizes of lower iron components (30 mm, 35 mm, and 40 mm) to investigate their effect on the generated magnetic field. As illustrated in Fig. 12g, increasing the length of the lower iron part correlates with enhanced magnetic field strength at a given point, especially at close distances. These findings indicate that the dimensions of the lower iron components can be strategically designed to optimize magnetic field generation for specific applications, meeting targeted operational requirements.

Another design parameter investigated in COMSOL simulations was the distance between the upper and lower parts of the structure of TME. The results indicated that this parameter had no significant effect on the generated magnetic field compared to other design parameters. The outcomes of this simulation study are presented in the Supplementary Materials (Figs. S9 and S10). Since the design objective was to make the end effector as compact and lightweight as possible, the distance between the upper and lower parts was minimized. Consequently, the focus for optimizing magnetic field generation was placed on other design parameters that exhibited greater influence on the field strength during both design and fabrication stages.

When employing a TME for a specific task, three critical parameters must be considered: the region of interest, magnetic field strength and gradient requirements, and the size and mobility of the TME. (1) Region of interest: this parameter defines the effective coverage area of the TME and the operational range in both OFF and ON states. (2) Magnetic field strength and gradient: the intensity and variation of the magnetic field produced within the workspace are essential for the effective performance of the magnetic robots. (3) Size and mobility: the dimensions and mobility of the TME must align with the setup and application requirements.

These parameters should be tailored to the specific task at hand. Simulations, as presented, are crucial for optimizing the design to effectively deliver a TME with suitable materials, dimensions, and magnetic field strength that meet application requirements. By analyzing the simulation outcomes, adjustments can be efficiently made to enhance performance, such as modifying the design of the magnets or the steel components to achieve optimal magnetic field generation. This approach not only saves time and resources during the fabrication process but also ensures that the TME is precisely engineered to meet the demands of its intended application.

## Supplementary information

Supplementary Tables, Figures, and References

Description of Additional Supplementary Files

Supplementary Video S1

Supplementary Video S2

Supplementary Video S3

Supplementary Video S4

Supplementary Video S5

Supplementary Video S6

Supplementary Video S7

Article File

## Data Availability

All data supporting the findings of this study are provided in the main article and the Supplementary Information. Additional raw datasets generated during the experiments cannot be shared publicly at this time due to ongoing intellectual property protection and a follow-up study currently in preparation. Deidentified or summary data may be made available from the corresponding author upon reasonable request and subject to institutional approval.

## Code availability

Custom scripts developed for data processing and analysis are available from the corresponding author upon reasonable request. Parts of the workflow rely on licensed software packages, and therefore, some project files cannot be publicly released. All parameters, algorithms, and procedures required to reproduce the results are provided in the main article and the Supplementary Information.

## References

[1] Lee, S. et al. A capsule-type microrobot with pick-and-drop motion for targeted drug and cell delivery. *Adv. Healthc. Mater.* **7**, e1700985 (2018). 29460365 10.1002/adhm.201700985

[2] Kim, N. G. et al. External steering of vine robots via magnetic actuation. *Soft Robotics* **12**, 159–170 (2024). 10.1089/soro.2023.0182 PMC12021788 39288083

[3] Abolfathi, K. et al. Independent and hybrid magnetic manipulation for full body controlled soft continuum robots. *IEEE Robot. Autom. Lett.* **8**, 4235–4242 (2023).

[4] Baburova, P. I. et al. Magnetic soft robot for minimally invasive urethral catheter biofilm eradication. *ACS Nano* **17**, 20925–20938 (2023). 37871301 10.1021/acsnano.2c10127

[5] Jeon, S. et al. A magnetically controlled soft microrobot steering a guidewire in a three-dimensional phantom vascular network. *Soft Robot.* **6**, 54–68 (2019). 30312145 10.1089/soro.2018.0019 PMC6386781

[6] Chen, Z. et al. A magnetic multi-layer soft robot for on-demand targeted adhesion. *Nat. Commun.* **15**, 644 (2024). 38245517 10.1038/s41467-024-44995-9 PMC10799857

[7] Ren, Z. & Sitti, M. Design and build of small-scale magnetic soft-bodied robots with multimodal locomotion. *Nat. Protoc.* **19**, 441–486 (2024). 38097687 10.1038/s41596-023-00916-6

[8] Mousavi, A., Ahmed, A., Khaksar, H., Choi, H. & Hoshiar, A. K. An input saturation-tolerant position control method for magnetic microrobots using adaptive fuzzy sliding-mode method. *IEEE Trans. Automation Sci. Eng.*10.1109/tase.2024.3400602 (2024).

[9] Abolfathi, K., Yazdi, M. R. H. & Hoshiar, A. K. Studies of different swarm modes for the MNPs under the rotating magnetic field. *IEEE Trans. Nanotechnol.* **19**, 849–855 (2020). vol.

[10] Lu, L. et al. Design and control of the magnetically actuated micro/nanorobot swarm toward biomedical applications. *Adv. Health. Mater.* **13**, e2400414 (2024). 10.1002/adhm.202400414 38412402

[11] Omisore, O. M. et al. A review on flexible robotic systems for minimally invasive surgery. *IEEE Trans. Syst. Man Cybern. Syst.* **52**, 631–644 (2022).

[12] Gong, D., Celi, N., Zhang, D. & Cai, J. Magnetic biohybrid microrobot multimers based on chlorella cells for enhanced targeted drug delivery. *ACS Appl. Mater. Interfaces* **14**, 6320–6330 (2022). 35020358 10.1021/acsami.1c16859

[13] Gundersen, R. A. et al. Generation of magnetic biohybrid microrobots based on MSC.sTRAIL for targeted stem cell delivery and treatment of cancer. *Cancer Nanotechnol.* **14**, 54 (2023). 37869575 10.1186/s12645-023-00203-9 PMC7615227

[14] Zhang, Y., Zhang, Y., Han, Y. & Gong, X. Micro/nanorobots for medical diagnosis and disease treatment. *Micromachines* **13**, 648 (2022). 10.3390/mi13050648 PMC9146405 35630115

[15] Nauber, R. et al. Medical microrobots in reproductive medicine from the bench to the clinic. *Nat. Commun.* **14**, 728 (2023). 36759511 10.1038/s41467-023-36215-7 PMC9911761

[16] Choi, J., Hwang, J., Kim, J. Y. & Choi, H. Recent progress in magnetically actuated microrobots for targeted delivery of therapeutic agents. *Adv. Health. Mater.* **10**, e2001596 (2021). 10.1002/adhm.202001596 33331143

[17] Park, J., Kim, J. Y., Pane, S., Nelson, B. J. & Choi, H. Acoustically mediated controlled drug release and targeted therapy with degradable 3D porous magnetic microrobots. *Adv. Health. Mater.* **10**, e2001096 (2021). 10.1002/adhm.202001096 33111498

[18] Mahoney, A. W. & Abbott, J. J. Five-degree-of-freedom manipulation of an untethered magnetic device in fluid using a single permanent magnet with application in stomach capsule endoscopy. * Int. J. Robot. Res.* **35**, 129–147 (2016).

[19] Nguyen, K. T. et al. A magnetically guided self-rolled microrobot for targeted drug delivery, real-time X-ray imaging, and microrobot retrieval. *Adv. Health. Mater.* **10**, e2001681 (2021). 10.1002/adhm.202001681 33506630

[20] Jeon, S. et al. A magnetically powered stem cell-based microrobot for minimally invasive stem cell delivery via the intranasal pathway in a mouse brain. *Adv. Health. Mater.* **10**, e2100801 (2021). 10.1002/adhm.202100801 34160909

[21] Hwang, J. et al. An electromagnetically controllable microrobotic interventional system for targeted, real-time cardiovascular intervention. *Adv. Health. Mater.* **11**, e2102529 (2022). 10.1002/adhm.202102529 35137568

[22] Ongaro, F., Pane, S., Scheggi, S. & Misra, S. Design of an electromagnetic setup for independent three-dimensional control of pairs of identical and nonidentical microrobots. *IEEE Trans. Robot.* **35**, 174–183 (2019).

[23] Sikorski, J., Heunis, C. M., Franco, F. & Misra, S. The ARMM system: an optimized mobile electromagnetic coil for non-linear actuation of flexible surgical instruments. *IEEE Trans. Magn.* **55**, 1–9 (2019).

[24] Yang, Z. et al. Magnetic control of a steerable guidewire under ultrasound guidance using mobile electromagnets. *IEEE Robot. Autom. Lett.* **6**, 1280–1287 (2021).

[25] Pittiglio, G. et al. Collaborative magnetic manipulation via two robotically actuated permanent magnets. *IEEE Trans. Robot.* **39**, 1407–1418 (2022).

[26] Brockdorff, M. et al. Hybrid trajectory planning of two permanent magnets for medical robotic applications. * Int. J. Robot. Res.* **44**, 273–290 (2025).

[27] Chen, R. & Folio, D. Electromagnetic actuation microrobotic systems. *Curr. Robot. Rep.* **3**, 119–126 (2022).

[28] Ryan, P. & Diller, E. Magnetic actuation for full dexterity microrobotic control using rotating permanent magnets. *IEEE Trans. Robot.* **33**, 1398–1409 (2017).

[29] Davy, J., Brockdorff, M. & Valdastri, P. Utilizing field gradient measurements for object tracking in permanent magnet based manipulation systems. *IEEE Trans. Magnet.* **40**, 1–1 (2025).

[30] McDonald, K. J., Kinnicutt, L., Moran, A. M. & Ranzani, T. Modulation of magnetorheological fluid flow in soft robots using electropermanent magnets. *IEEE Robot. Autom. Lett.* **7**, 3914–3921 (2022).

[31] Tavakoli, M., Viegas, C., Romao, J. C., Neto, P. & de Almeida, A. T. Switchable magnets for robotics applications. In *2015 IEEE/RSJ International Conference on Intelligent Robots and Systems (IROS**)* 4325–4330 (IEEE, 2025).

[32] Peidró, A., Tavakoli, M., Marín, J. M. & Reinoso, Ó Design of compact switchable magnetic grippers for the HyReCRo structure-climbing robot. *Mechatronics* **59**, 199–212 (2019).

[33] Zhang, L., Ji, X., Jiao, Y., Huang, Y. & Qian, H. Design and control of the “transboat”: a transformable unmanned surface vehicle for overwater construction. *IEEE/ASME Trans. Mechatron.* **28**, 1116–1126 (2022).

[34] Cheong, D., Park, H. & Kim, N. Design and maneuver of a tool-changer using switchable magnet for a tool hung by a cable. In *2024 IEEE 20th International Conference on Automation Science and Engineering (CASE**)* 1252–1257 (IEEE, 2024).

[35] Kim, D., Butler, C. & Costello, M. Rotorcraft robotic landing gear with locking mechanisms. *J. Dyn. Syst. Meas. Control* **145**, 061002 (2023).

[36] Dalton, D. K., Tabor, G. F., Hermans, T. & Abbott, J. J. Position regulation of a conductive nonmagnetic object with two stationary rotating-magnetic-dipole field sources. *IEEE Trans. Robot.* **40**, 4635–4647 (2024).

[37] Son, D., Ugurlu, M. C. & Sitti, M. Permanent magnet array-driven navigation of wireless millirobots inside soft tissues. *Sci. Adv.* **7**, eabi8932 (2021). 34669466 10.1126/sciadv.abi8932 PMC8528412

[38] Kim, Y., Parada, G. A., Liu, S. & Zhao, X. Ferromagnetic soft continuum robots. *Sci. Robot.* **4**, eaax7329 (2019). 33137788 10.1126/scirobotics.aax7329

[39] Lloyd, P. et al. A learnt approach for the design of magnetically actuated shape forming soft tentacle robots. *IEEE Robot. Autom. Lett.* **5**, 3937–3944 (2020).

[40] Pittiglio, G. et al. Patient-specific magnetic catheters for atraumatic autonomous endoscopy. *Soft Robot.* **9**, 1120–1133 (2022). 35312350 10.1089/soro.2021.0090 PMC9805888

[41] G. Pittiglio, G. et al. Personalized magnetic tentacles for targeted photothermal cancer therapy in peripheral lungs. *Commun. Eng.*10.1038/s44172-023-00098-9 (2023).

[42] Masiero, F. & Sinibaldi, E. Exact and computationally robust solutions for cylindrical magnets systems with programmable magnetization. *Adv. Sci.* **10**, e2301033 (2023). 10.1002/advs.202301033 PMC10477869 37460392

[43] Reich, F. A., Stahn, O. & Müller, W. H. The magnetic field of a permanent hollow cylindrical magnet. *Contin. Mech. Thermodyn.* **28**, 1435–1444 (2015).

[44] Camacho, J. M. & Sosa, V. Alternative method to calculate the magnetic field of permanent magnets with azimuthal symmetry. *Rev. Mexicana de. FíScia E* **59**, 8–17 (2013).

