# Supplementary material for: Magnetic field control with dual robotic tunable magnetic end effectors
Source: Commun Eng. 2026 Mar 4;5:68. doi: 10.1038/s44172-026-00629-0 (PMC13077044; doi:10.1038/s44172-026-00629-0)
Supplement: Supplementary file 1 — Supplementary Tables, Figures, and References [file 44172_2026_629_MOESM1_ESM.pdf]

Supplementary Information for:

Magnetic Field Control with Dual Robotic Tunable Magnetic End Effectors

Kiana Abolfathi[1], Jiacheng Zhu[1], James H. Chandler[2], Keyoumars Ashkan[3], Pietro Valdastri[2], Hongsoo Choi[4, 5], Xiaojun Zhai[1], and Ali Kafash Hoshiar[1]*

[1] School of Computer Science and Electronic Engineering, University of Essex, Colchester, UK

[2] STORM Lab, Institute of Robotics, Autonomous Systems and Sensing, School of Electronic and Electrical Engineering, University of Leeds, Leeds, U.K.

[3] Department of Neurosurgery, King's College Hospital, London, U.K.

[4] Department of Robotics and Mechatronics Engineering and the DGIST-ETH Microrobotics Research Center, Daegu Gyeongbuk Institute of Science and Technology, Daegu South Korea

[5] Department of Biomedical Engineering, Institute for Applied Life Sciences, University of Massachusetts Amherst, 240 Thatcher Road, Amherst, MA 01003, USA

E-mail: a.kafashhoshiar@essex.ac.uk, [*]corresponding author

This document contains supplementary figures, tables, and references that support the main manuscript.

Supplementary Tables

Table S1: Comparison of magnetic field generation systems.

Study	Maximum Magnetic Field Strength	Workspace Area	Magnetic Field Gradient	Frequency Range	DOF
[1]	94 mT<br>(m = 12.7 $A.m^2$and r = 30 mm)	22.2 mm diameter of the lumen	Not applicable	0.04-0.8 Hz	6
[2]	64 mT<br>(m = 40 $A.m^2$ and r = 50 mm)	±50 mm from the center	Not applicable	15 Hz	3
[3]	200 mT	0.1 * 0.1* 0.1 m^3	0.5 $\frac{T}{m}$	Not applicable	8 and far distances 9
[4]	Not applicable	Circle with radius 30 mm	7 $\frac{T}{m}$	Not applicable	3
[5]	30 mT	Spherical region with 5 mm diameter	0.83 $\frac{T}{m}$	0 to 1.6 Hz	5
TME	12 mT<br>(it is changeable by using different design)	Sphere with radius 0.62 m (CR 3 robotic arm workspace)	0-0.86 $\frac{T}{m}$	Not used in this study	7

Table S2: Functional comparison between prior PM-based magnetic systems and the proposed dual TME.

Study	Is workspace always under active magnetic field?	Magnetic field strength changeable without actuator movement?	Able to generate different magnetic field regions without actuator movement?	Is there any ON/OFF mode?	Directional field modulation without moving actuator?	Changeable position of PMs?
[1]	**Yes**<br>Rotating fields always generate magnetic fields and there is no OFF mode	**Yes**<br>The magnetic field strength changes as the PM rotates relative to the untethered magnetic robot	**Yes**<br>Rotating the PM axis creates different magnetic field regions, as shown by analytical dipole mapping	**No**<br>Cannot turn off the PM	**Yes**<br>Magnetic field direction can be controlled by rotating the PM	**Yes**<br>6 DOF robotic arm.
[2]	**Yes**<br>Both rotating PM dipoles continuously generate rotating magnetic fields	**No**<br>Magnetic field strength is not tunable by rotation of PM	**Yes**<br>Changing rotation axis generates different magnetic field regions without actuator motion	**No**<br>Rotating PMs always generate magnetic fields	**Yes**<br>The rotation axis vector is changed	**No**<br>Stationary position of the PMs
[3]	**Yes**<br>External robotically PMs continuously generate a magnetic field. Workspace is always under magnetic effect	**No**<br>Magnetic field strength control requires spatial repositioning of the robot arms	**No**<br>Different regions require moving one or both PMs, not achievable in a fixed configuration	**No**<br>Magnetic field cannot be canceled without moving PM far away by robotic arm movement	**Limited**<br>Magnetic field direction can change by arm repositioning orientation	**Yes**<br>Robotic arm for movement of the PMs
[4]	**Yes**<br>The 100-magnet array creates a constant force trap around the workspace. Always active	**No**<br>Magnetic field strength fixed by magnet array geometry	**No**<br>Region is fixed by array geometry	**No**<br>No method to cancel the static magnetic field	**No**<br>Field orientation is fixed relative to the array unless the array is physically moved	**No**<br>Fix array
[5]	**Yes**<br>Rotating PM always generates magnetic fields	**No**<br>Rotation changes direction of the magnetic field	**Limited**<br>Can shape magnetic fields but cannot isolate different workspace regions independently	**Limited**<br>There is no OFF mode. But in some conditions able to zero-field state	**Yes**<br>Each PM can rotate independently	**No**<br>PM control rotation does not position
**Dual TME**	**No**<br>Internal θ changes, achieves an OFF state with no active magnetic field effect on the workspace	**Yes**<br>Magnetic field strength tunable via internal θ rotation and α adjustments without arm repositioning	**Yes**<br>Dual TME can activate different workspace regions independently while stationary.	**Yes**<br>By rotating its internal structure	**Yes**<br>Directional modulation achieved via internal α rotation in the TME	**Yes**<br>Robotic arm for movement of the TME

Table S3: Structural parameters of the TME for generating different magnetic field strengths at 15 mm.

		**Figure S7(a)**	**Figure S7(b)**	**Figure S7(c)**
**Upper Assembly**	Number of the PMs	4	4	4
	Material of the PMs	NdFeB	NdFeB	NdFeB
	Size of the PMs (diameter * length)	8 * 25 mm	12 * 25 mm	20 * 40 mm
	Iron Parts Sizes	30 * 24 * 5 mm	40 * 40 * 5 mm	40 * 55 * 5 mm
	Iron Parts Material	EN3B mild steel	EN3B mild steel	EN3B mild steel
**Lower Assembly**	Number of the PMs	2	2	2
	Material of the PMs	NdFeB	NdFeB	NdFeB
	Size of the PMs (diameter * length)	8 * 25 mm	12 * 25 mm	20 * 40 mm
	Iron Parts Sizes	35 * 13 * 5 mm	40 * 20 * 5 mm	55 * 30 * 5 mm
	Iron Parts Material	EN3B mild steel	EN3B mild steel	EN3B mild steel
	Maximum Magnetic Field at 15 mm distance in x direction	12.3 mT	20.3 mT	45.7 mT

Table S4: TME structural configurations for generating up to 12 mT at various distances.

		**Figure S8(a)**	**Figure S8(b)**
**Upper Assembly**	Number of the PMs	4	4
	Material of the PMs	NdFeB	NdFeB
	Size of the PMs (diameter * length)	8 * 25 mm	20 * 40 mm
	Iron Parts Sizes	30 * 24 * 5 mm	40 * 55 * 5 mm
	Iron Parts Material	EN3B mild steel	EN3B mild steel
**Lower Assembly**	Number of the PMs	2	2
	Material of the PMs	NdFeB	NdFeB
	Size of the PMs (diameter * length)	8 * 25 mm	20 * 40 mm
	Iron Parts Sizes	35 * 13 * 5 mm	55 * 30 * 5 mm
	Iron Parts Material	EN3B mild steel	EN3B mild steel
	The distance at which the TME can generate approximately 12 mT in the ON condition.	15 mm	45 mm

Supplementary Figures

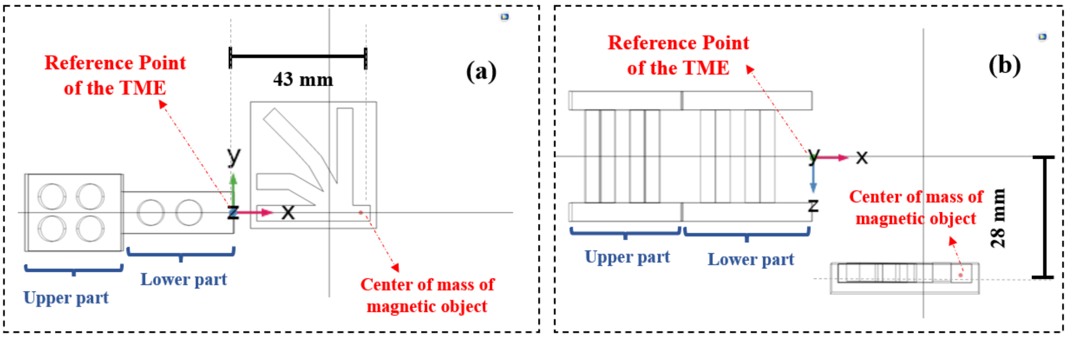

Figure S1: Position of the TME (excluding outer casing and motor) relative to the phantom and the magnetic milli-carrier, as predicted by the ANN for the Junction 1 path steering based on the direction of motion and required magnetic field strength. (a)Y-X View (b) X-Z View.

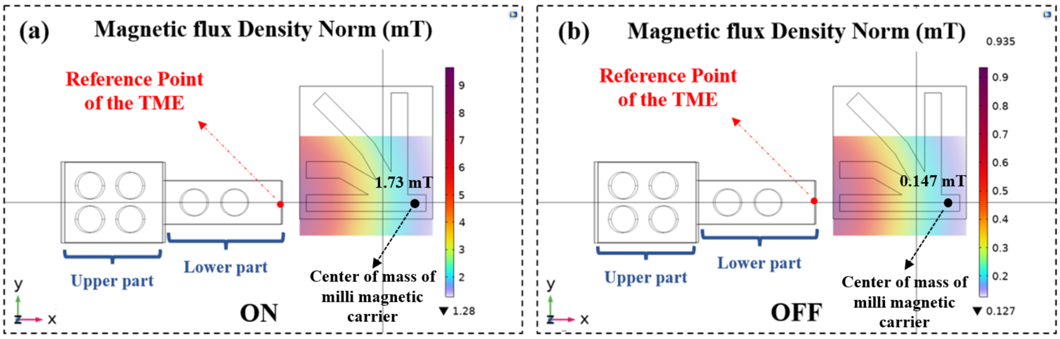

Figure S2: Magnetic flux density norm plots for the on and off conditions of the TME. (a) TME is ON. (b) TME is OFF.

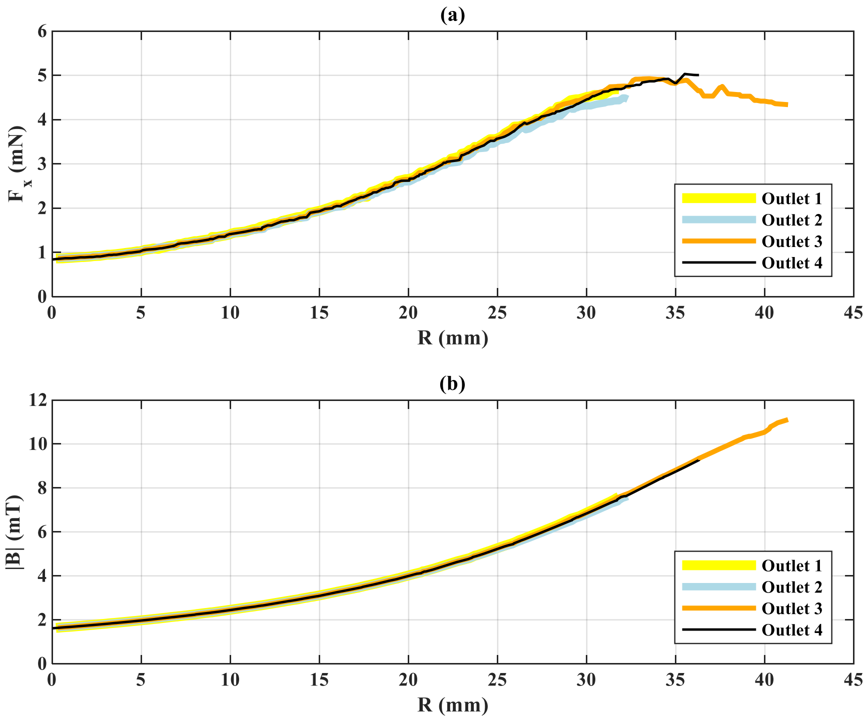

Figure S3: Magnetic force range of the milli magnetic carrier across four outlets steered by the TME. R denotes the distance of the carrier's center of mass from its starting point.

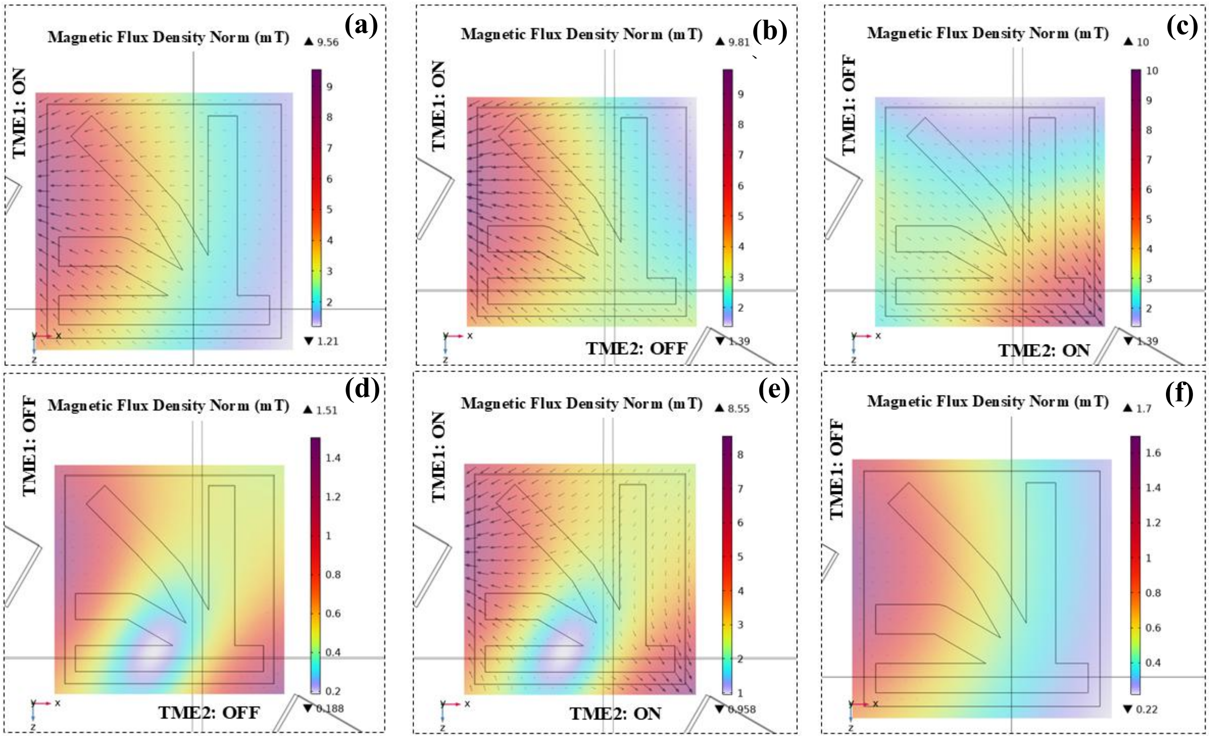

Figure S4: Magnetic field generated in different region in start point to outlet junction 2 by dual TME.

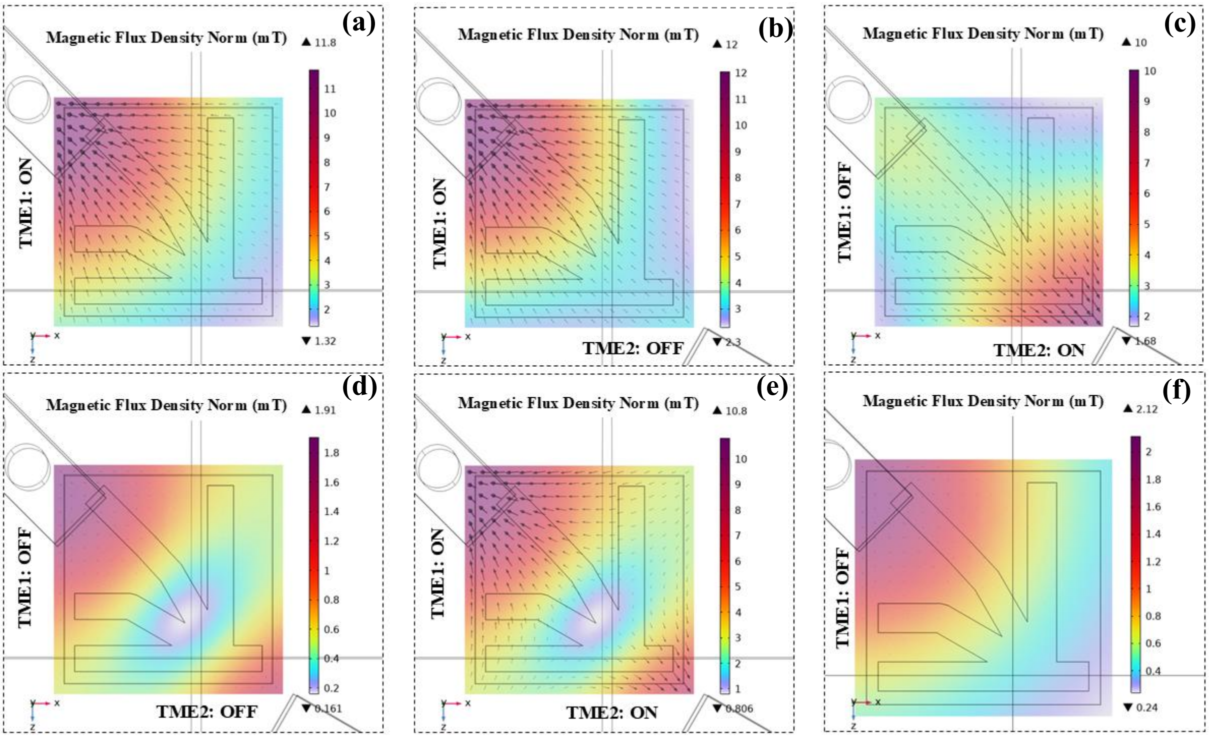

Figure S5: Magnetic field generated in different region in start point to outlet junction 3 by dual TME.

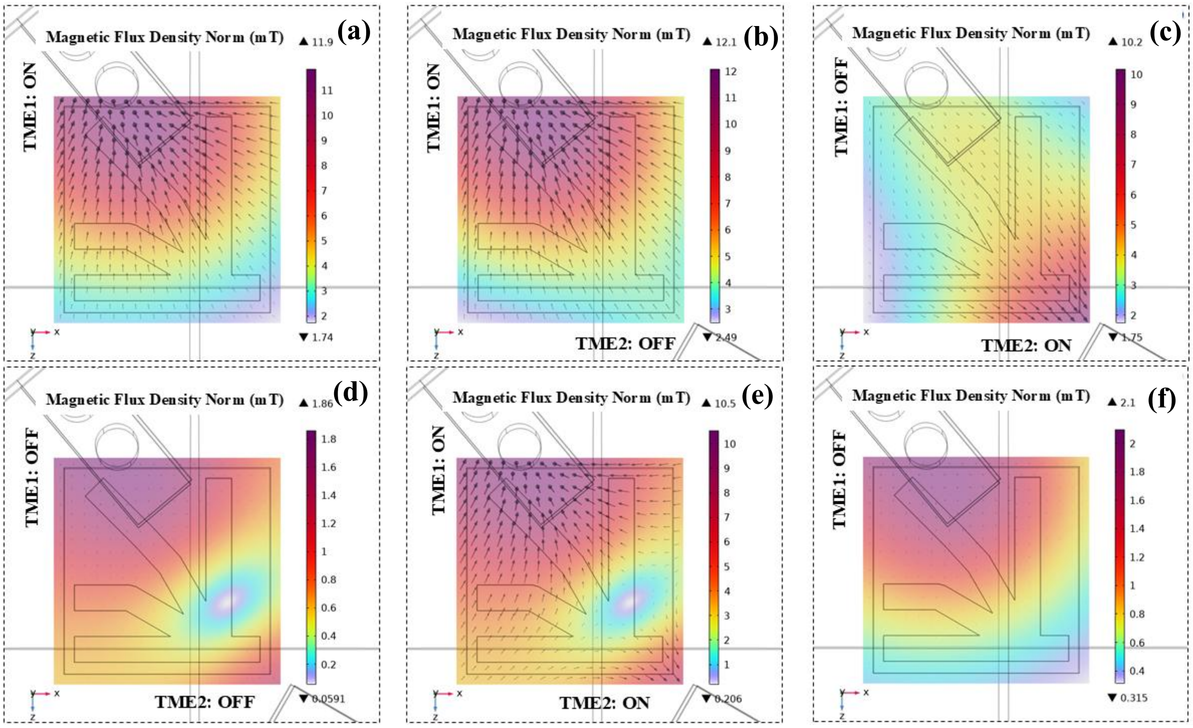

Figure S6: Magnetic field generated in different region in start point to outlet junction 4 by dual TME.

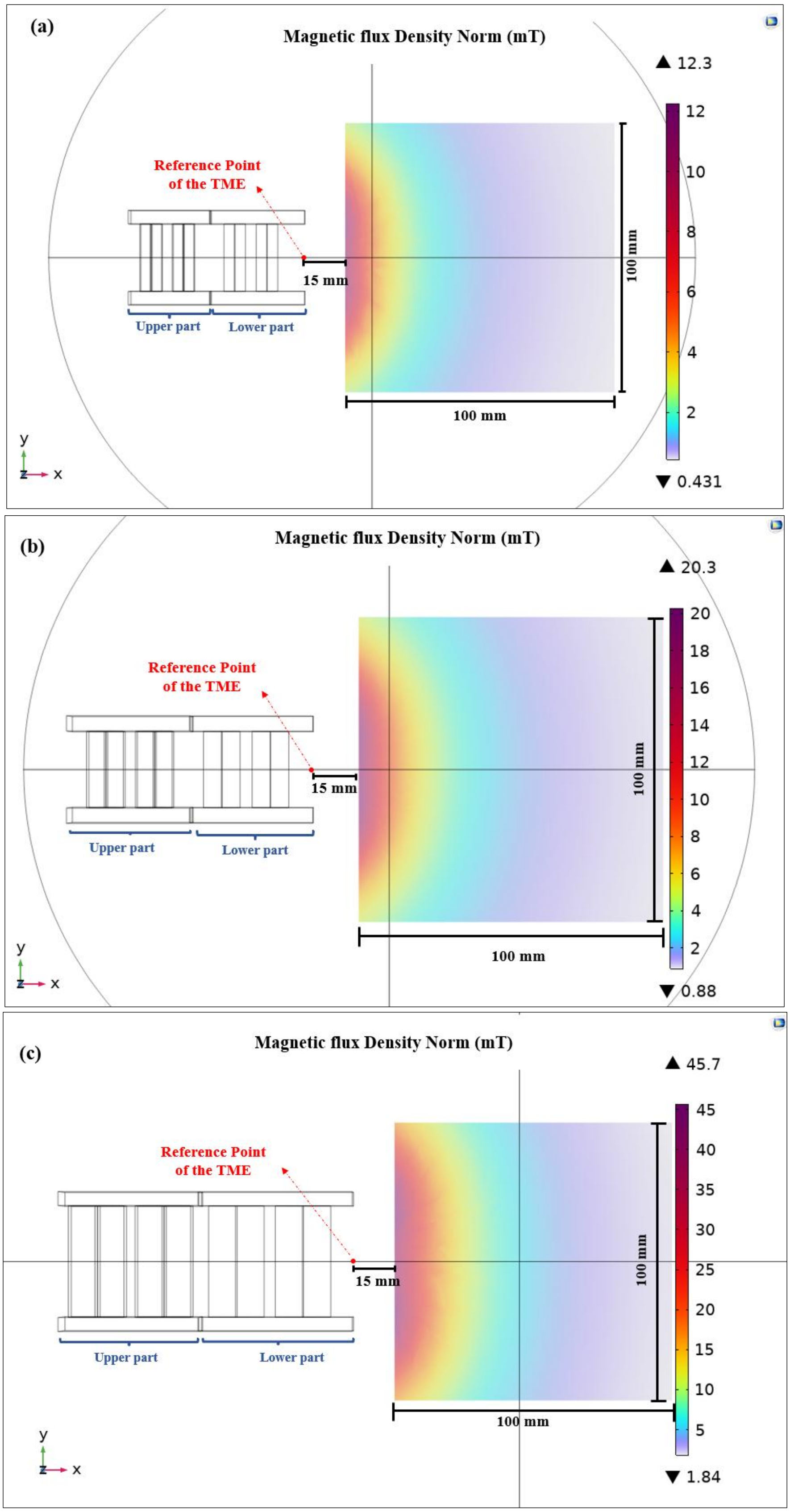

Figure S7: Simulated Magnetic Field Distributions at 15 mm for Various TME Designs Using COMSOL. Subfigures (a)–(c) present results for different internal structure sizes of the TME, with the dimensions of each design reported in Table S3.

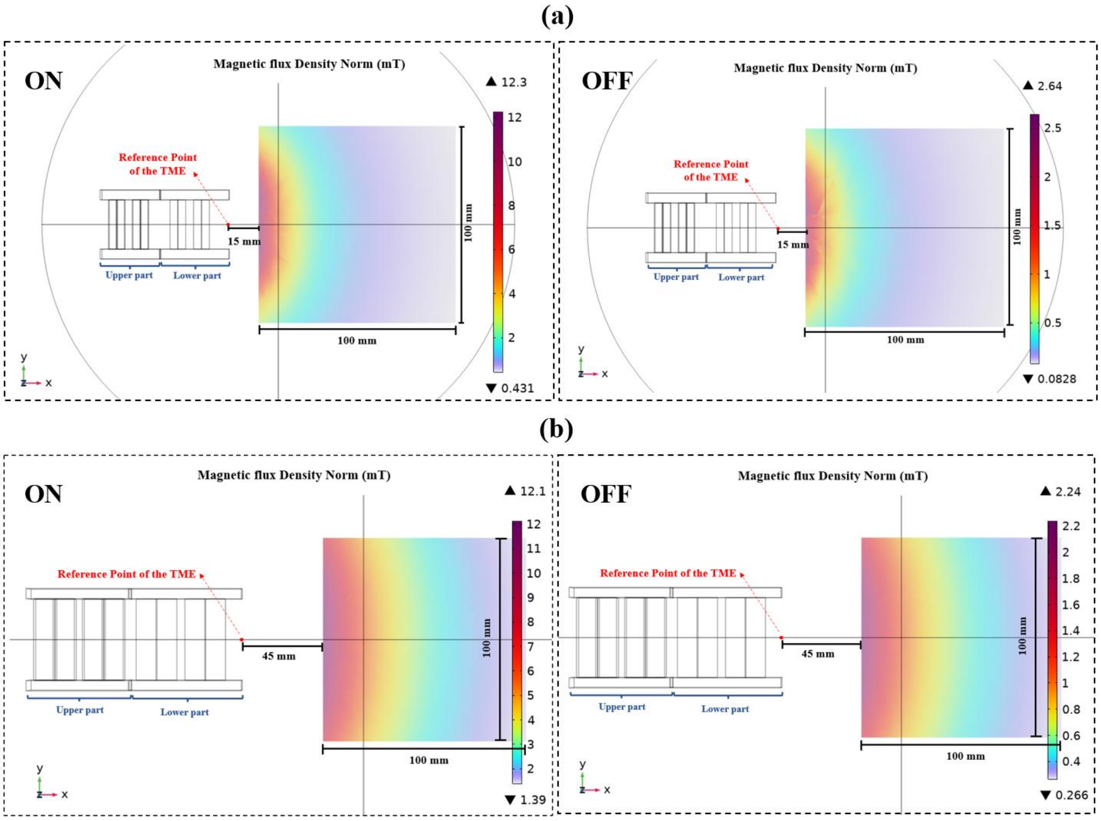

Figure S8: Simulated magnetic field distributions for achieving 12 mT at various distances within the same workspace volume. (a) Distribution at 15 mm (along the x direction) from the TME reference point. (b) Distribution at 45 mm (along with the x direction) from the TME reference point. The dimensions of each design are reported in Table S4.

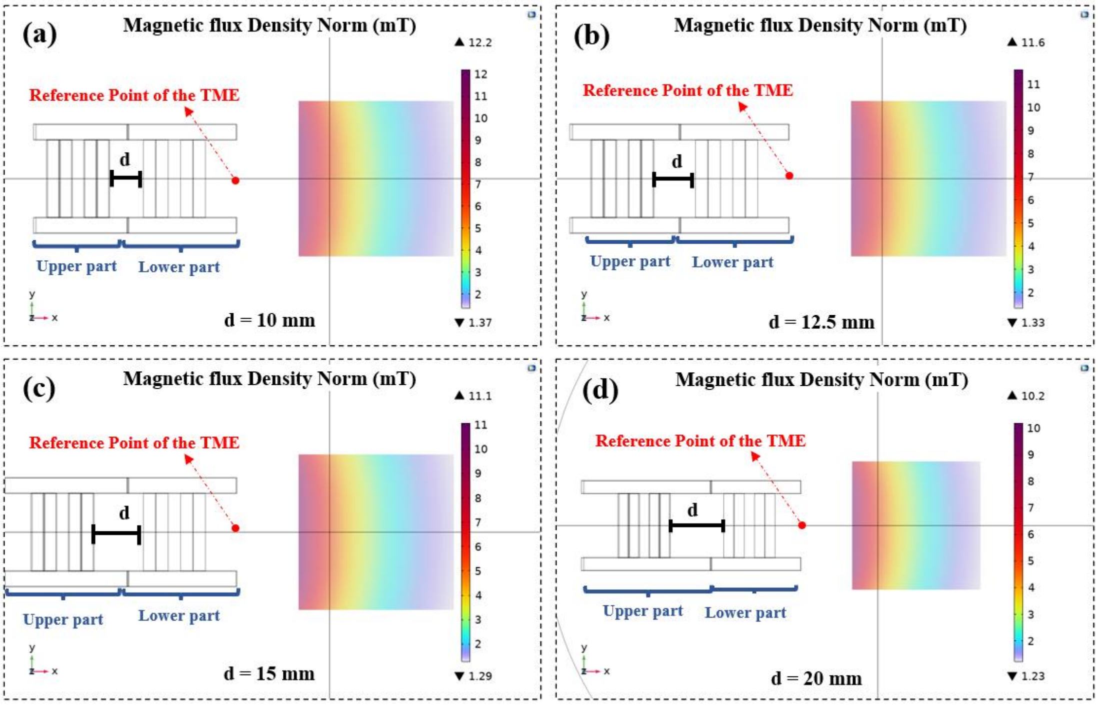

Figure S9: COMSOL simulations showing the effect of varying the distance (d) between the upper and lower parts of the TME on magnetic flux density at the reference point, for distances of (a) 10 mm, (b) 12.5 mm, (c) 15 mm, and (d) 20 mm.

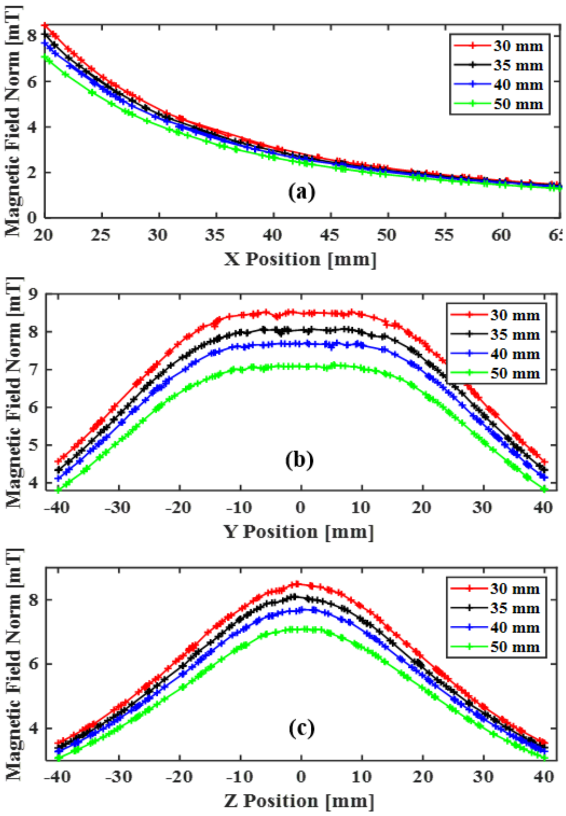

Figure S10: Simulated magnetic field distribution along the (a) X, (b) Y, and (c) Z axes for different distances between the upper and lower parts of the TME (30, 35, 40, and 50 mm).

Supplementary References

[1] A. W. Mahoney and J. J. Abbott, "Generating Rotating Magnetic Fields With a Single Permanent Magnet for Propulsion of Untethered Magnetic Devices in a Lumen," *IEEE Transactions on Robotics,* vol. 30, no. 2, pp. 411-420, 2014, doi: 10.1109/tro.2013.2289019.

[2] D. K. Dalton, G. F. Tabor, T. Hermans, and J. J. Abbott, "Position Regulation of a Conductive Nonmagnetic Object With Two Stationary Rotating-Magnetic-Dipole Field Sources," *IEEE Transactions on Robotics,* vol. 40, pp. 4635-4647, 2024, doi: 10.1109/tro.2024.3454568.

[3] G. Pittiglio, M. Brockdorff, T. da Veiga, J. Davy, J. H. Chandler, and P. Valdastri, "Collaborative Magnetic Manipulation via Two Robotically Actuated Permanent Magnets," *IEEE Transactions on Robotics,* vol. 39, no. 2, pp. 1407-1418, 2023, doi: 10.1109/tro.2022.3209038.

[4] D. Son, M. C. Ugurlu, and M. Sitti, "Permanent magnet array–driven navigation of wireless millirobots inside soft tissues," *Science Advances,* vol. 7, no. 43, p. eabi8932, 2021.

[5] P. Ryan and E. Diller, "Magnetic Actuation for Full Dexterity Microrobotic Control Using Rotating Permanent Magnets," *IEEE Transactions on Robotics,* vol. 33, no. 6, pp. 1398-1409, 2017, doi: 10.1109/tro.2017.2719687.
