# Supplementary material for: Magnetic field control with dual robotic tunable magnetic end effectors
Source: Commun Eng. 2026 Mar 4;5:68. doi: 10.1038/s44172-026-00629-0 (PMC13077044; doi:10.1038/s44172-026-00629-0)
Supplement: Supplementary file 2 — Description of Additional Supplementary Files [file 44172_2026_629_MOESM2_ESM.pdf]

Description of Additional Supplementary Files:

File name: Supplementary Video S1

Description: Magnetic field data collection using the Tunable magnetic end effector (TME) and a 3-axis magnetic sensor mounted on the robotic arm, showing the experimental measurement process and data collection.

File name: Supplementary Video S2

Description: 3D visualization of the magnetic field distribution under ON and OFF states of the Tunable magnetic end effector (TME), illustrating spatial variations in the workspace.

File name: Supplementary Video S3

Description: ANN-based magnetic steering of a milli magnetic carrier using dual Tunable magnetic end effectors (TMEs) for controlled navigation through predefined paths.

File name: Supplementary Video S4

Description: Dynamic switching of magnetic field regions using dual Tunable magnetic end effectors (TMEs), demonstrated through combined experimental results and simulations.

File name: Supplementary Video S5

Description: Shape formation of a magnetic soft robot with and without the memory effect using the Tunable magnetic end effector (TME), highlighting the influence of actuation history.

File name: Supplementary Video S6

Description: Magnetic field direction control using dual Tunable magnetic end effectors (TMEs) to expand bending range and enable multiple shape formations of a soft robot.

File name: Supplementary Video S7

Description: ANN-guided steering of magnetic nanoparticle swarms using the Tunable magnetic end effector (TME) to control speed and direction through junctions.
